# Sirtuin1 mitigation of calcium oxalate nephropathy via enhancing itaconate abundance through reduction of histone trimethylation

**DOI:** 10.1002/ctm2.70450

**Published:** 2025-08-18

**Authors:** Xiangyang Yao, Haoran Liu, Chen Duan, Yangjun Zhang, Xiaoliang Wu, Bo Li, Sheng Li, Yan Gong, Tongzu Liu, Xinghuan Wang, Hua Xu

**Affiliations:** ^1^ Department of Urology Zhongnan Hospital of Wuhan University Wuhan China; ^2^ Department of Urology The First Affiliated Hospital of Anhui Medical University Hefei China; ^3^ Department of Urology The First Affiliated Hospital of University of Science and Technology Hefei China; ^4^ Department of Urology The First Affiliated Hospital of Zhengzhou University Henan China; ^5^ Tumor Precision Diagnosis and Treatment Technology and Translational Medicine Hubei Engineering Research Center Zhongnan Hospital of Wuhan University Wuhan China; ^6^ Taikang Center for Life and Medical Sciences Wuhan University Wuhan China

**Keywords:** calcium oxalate nephropathy, itaconate, macrophage, Sirt1

## Abstract

**Background:**

Clinical therapeutic approaches to prevent and treat renal injury in patients with acute kidney injury (AKI) and chronic kidney disease (CKD) induced by calcium oxalate (CaOx) are limited. As a pivotal deacetylase, Sirtuin1 (Sirt1) exhibits notably anti‐inflammatory effects, but its metabolic mechanism in regulating CaOx nephropathy remains unexplored.

**Methods:**

We analysed organic acid metabolism in kidney using the nontargeted metabolome and identified key targets by RNA‐seq. Evaluate renal injury and oxidative stress using techniques such as Positron Emission Tomography‐Computed Tomography (PET/CT) and transmission electron microscope. The protective mechanisms of Sirt1 against CaOx‐induced kidney injury and subsequent crystal deposition were demonstrated using in vitro coculture systems and in vivo Sirt1 conditional knockout mice.

**Results:**

We found that Sirt1 has a significant protective effect on renal injury and oxidative stress induced by CaOx. Sirt1 expression decreases in CaOx nephropathy mice, and activation of Sirt1 reduces CaOx‐induced kidney injury and crystal deposition by increasing the level of itaconate. In addition, it was found that Sirt1 enhances immunoresponsive gene 1 and inhibits Sdha by trimethylating histones, thereby regulating the oxidation levels of itaconate and succinate. Furthermore, we emphasise the valuable role of Sirt1 agonists and exogenous itaconate in alleviating crystal induced kidney injury.

**Conclusions:**

Our study revealed a previously unknown function of Sirt1 in CaOx nephropathy. By regulating itaconate level through epigenetic, Sirt1 protects against renal inflammation and oxidative damage induced by CaOx. Our preclinical data suggest that targeted Sirt1 agonism represents a promising therapeutic intervention for progressive crystallopathic nephropathy, potentially disrupting the inflammation–crystallisation vicious cycle.

**Highlights:**

- Sirt1 is significantly reduced in CaOx‐induced nephropathy models in vivo and calcium oxalate monohydrate (COM) induced in vitro.
- Sirt1 in macrophages alleviated CaOx‐induced kidney injury and crystal deposition via elevating itaconate levels. Conditional knockout of Sirt1 in vivo significantly exacerbates renal crystal deposition and function damage.
- We accurately evaluated CaOx‐induced renal inflammation status by micro ^18^F‐FDG PET/CT, and observed macrophage phagocytosis and encapsulation of crystals exposed to epithelial cells through scanning electron microscopy.
- Sirt1 agonists can be used as preventative and therapeutic agents for CaOx nephropathy, and exogenous supplementation of itaconate effectively alleviated renal crystallisation and inflammatory damage.

## Graphical abstract

Sirt1 alleviated CaOx‐induced kidney injury and crystal deposition via elevating itaconate levels. Conditional knockout of Sirt1 in vivo significantly exacerbates renal crystal deposition and function damage. Exogenous supplementation of itaconate effectively alleviated renal crystallisation and inflammatory damage.

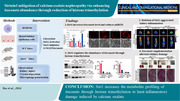

## 1 INTRODUCTION

Renal damage instigated by crystals within the kidney serves as a critical factor in the development and progression of nephrocalcinosis, acute kidney injury (AKI) and chronic kidney disease (CKD).^1^, ^2^, ^3^ Crystal‐induced nephropathy is categorised into three types based on the crystal deposition site: intravascular (type 1), nephron (type 2) and urinary tract (type 3).^2^ The AKI caused by calcium oxalate (CaOx) crystals epitomises the classical manifestation of type 2 crystal‐induced AKI.^4^, ^5^ The presence of CaOx in renal tubules leads to its direct adherence to the tubular epithelial cells (TECs), triggering severe inflammatory responses and cellular damage, with macrophages (Mϕs) playing an indispensable role in this process.^6^, ^7^ It is understood that M1 macrophages (M1Mϕs) exacerbate crystal deposition by inducing inflammation‐related oxidative stress, whereas M2 macrophages (M2Mϕs) exhibit anti‐inflammatory properties and facilitate crystal phagocytosis.^8^, ^9^, ^10^ This phenotypic transformation is fundamentally driven by metabolic reprogramming.^11^ The alteration of the tricarboxylic acid (TCA) cycle represents a metabolic adaptation mechanism that accompanies the activation of inflammatory macrophages, where endogenous metabolites exert a regulatory influence on the inflammatory response.^11^

Succinate, a central metabolic intermediate at the interface of the TCA cycle and mitochondrial electron transport chain, serves dual regulatory functions as both a bioenergetic substrate for complex II (succinate dehydrogenase, SDH) and an inflammatory signalling molecule through IL‐1β‐mediated pathways.^12^, ^13^ Mitochondrial SDH generates abundant reactive oxygen species (ROS) during succinate oxidation, which stabilises the hypoxia‐inducible factor (HIF)‐1α through redox signals and promotes the release of IL‐1β.^11^, ^14^, ^15^ Conversely, itaconate, another metabolite in the TCA cycle, exhibits anti‐inflammatory properties in reducing ROS production by inhibiting SDH activity.^16^ Itaconate, a metabolite produced by the enzyme encoded by immunoresponsive gene 1 (Irg‐1), exhibits increased levels in lipopolysaccharide ‐activated Mϕs, eliciting antioxidant and anti‐inflammatory responses.^17^ Previous studies have demonstrated that pro‐inflammatory macrophages predominantly rely on glycolysis, generating more ROS through the oxidation of succinate by SDH. The inhibition of succinate oxidation can alter the pro‐inflammatory state of macrophages.^11^ Therefore, itaconate may be a valuable metabolic intermediate in regulating CaOx nephropathy.

Sirtuin1 (Sirt1), an NAD^+^‐dependent histone deacetylase, is essential in regulating cellular energy homeostasis, cell metabolism and aging.^18^ Studies have shown that Sirt1 is significantly up‐regulated during macrophage differentiation, where its NAD⁺‐dependent deacetylase activity mediates the silencing of inflammatory genes and restricts the expression of pro‐inflammatory genes.^19^ Our previous research indicated that metformin orchestrates macrophage polarisation towards the anti‐inflammatory M2 phenotype via Sirt1 activation, and this pharmacological reprogramming attenuated crystal deposition and kidney injury.^20^ Meanwhile, Sirt1 also inhibits the TLR4 and ferroptosis signalling pathway, thereby reducing inflammation and oxidative damage of TECs.^21^, ^22^ Consequently, we hypothesised that Sirt1 may impact the occurrence and progression of CaOx nephropathy by regulating macrophage metabolism.

Our results underscore that Sirt1 affects itaconate metabolism and succinate oxidation through the Irg‐1/Sdha pathway. This process mitigates inflammation and oxidative stress‐induced damage to TECs while alleviating CaOx crystal deposition. Sirt1 agonists might be a valuable option to delay progressive forms of CaOx nephropathy.

## 2 MATERIALS AND METHODS

### 2.1 Animal experiments

C57BL/6J mice (6–8 weeks) were obtained from the Hubei Experimental Animal Research Center. Myeloid conditional Sirt1 knockout mice generated by hybridising Sirt1^flox/flox^ mice (T006657; GemPharmatech Inc, China) and Lysm‐cre transgenic mice (T003822; GemPharmatech Inc, China). All mice were raised in the Center of Experimental Animals at Zhongnan Hospital of Wuhan University in accordance with the NIH Laboratory Animal Care and Use Guidelines (details can be found in our previous manuscript).^20^, ^23^ Groups of 6‐week‐old (*n* = 6 mice/group) male mice were randomised to receive intraperitoneally injected with glyoxylate (Gly) (100 mg/kg/d, 100 µL) continuously for 7 days to establish a CaOx nephropathy model,^10^, ^24^, ^25^ while the control mice were replaced with normal saline (100 mg/kg/d, 100 µL). To investigate the effect of Sirt1 on CaOx nephropathy, SRT1720 (20 mg/kg/d, 100 µL) and 4‐octyl itaconate (OI, 50 mg/kg/d, 100 µL) were injected intraperitoneally for 10 days,^26^, ^27^ and other drugs were injected intraperitoneally at 10 mg/kg/d. After 10 days, all animals were euthanised, and kidney tissues were collected. All animal experiments were approved by the Ethics Committee of Zhongnan Hospital of Wuhan University (MRI2023‐LACA18).

### 2.2 Primary cell culture

After mice were euthanised, the tibias and femurs of the hind limbs were excised. Bone marrow‐derived macrophages (BMDMs) were obtained by washing the bone marrow cavity with sterile PBS, and then incubated in RPMI 1640 medium containing 10% FBS and 50 ng/mL M‐CSF at 37°C and 5% CO_2_ for 7 days.

Meanwhile, we separated the kidney tissues of mice and crushed the renal cortex. After 30 min of digestion with type I collagenase (37°C), renal TECs were separated by gradient density centrifugation. To further investigate the effect of COM‐stimulated TECs on BMDMs, we simulated a BMDM‐TEC coculture system using a Transwell chamber.

### 2.3 Statistical analysis

All statistical analyses were performed with R software (version 4.1.1) and GraphPad Prism (version 8.0.2). The expression data are expressed as the mean ± SD. Student's *t*‐test and one‐way analysis of variance were used to compare group differences. *p* < .05 indicated statistical significance.

### 2.4 Additional methods

For details, please see Supporting Information.

## 3 RESULTS

### 3.1 Sirt1 enhances itaconate abundance and Irg‐1 levels in macrophages

**FIGURE 1:**
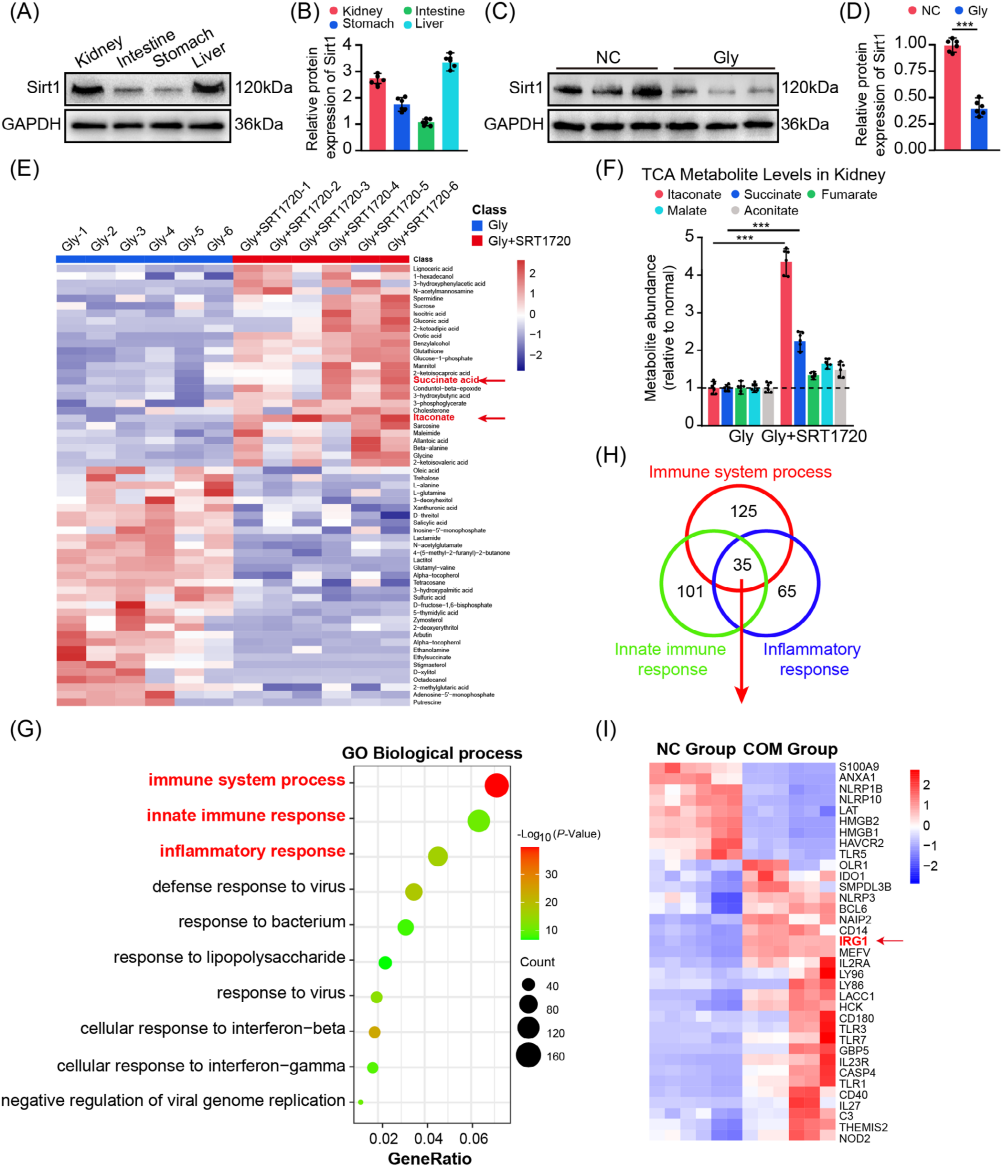
Sirt1 regulates organic acid metabolism in the kidneys of CaOx stones. (A and B) Western blot analysis of the expression of Sirt1 in major metabolic organs, including the liver, kidney, intestine and stomach (*n* = 6). (C and D) Western blot analysis of Sirt1 levels in renal tissues of different treatments, including negative control (NC) andGly (*n* = 3). (E) Identification of Sirt1 regulated renal organic acid metabolites by nontargeted metabonomic analysis (*n* = 6). (F) Changes in TCA metabolite levels in mouse kidneys (mainly changes in five organic acids). (G) Gene Ontology analysis of the biological processes of differentially expressed genes. (H) Venn diagram identified common differentially expressed genes in three pathways. (I) Heatmap indicating the expression panel of differentially expressed genes (*n* = 6). *p < .05, **p < .01, ***p < .001.

To delineate the organ‐specific expression profile of Sirt1 in mice, we quantified the mRNA and protein levels of Sirt1 in primary organs responsible for metabolism (including liver, kidney, intestine and stomach) of adult mice and found that its expression was higher in the kidney (Figures 1A,B and S1A). Additionally, a notable decrease in Sirt1 protein was observed in nephropathy mice induced by Gly (Figures 1C,D and S1B). However, administration of SRT1720—a pharmacological activator of Sirt1—did not elevate Sirt1 protein abundance (Figure S1C,D), since it mainly enhances Sirt1 enzymatic activity (Figure S1C,D). To assess the functional consequences of Sirt1 on organic acid metabolism in CaOx nephropathy mice, we performed untargeted GC–MS metabolomic analysis comparing the Gly group and Gly+SRT1720 group (Figure S1E). The results indicated significant accumulation of both itaconate and succinate upon Sirt1 activation (Figure 1E). In addition, we analysed the abundance of major metabolites in the TCA and found that the level of itaconate increased more than fourfold after Sirt1 activation and was the sole mitochondrial feature that occurred universally in a range of metabolically diverse tissues (Figure 1F). However, the increased level of succinate might be attributed to itaconate inhibiting succinate oxidation by SDH, leading to succinate accumulation. Therefore, we focused on the potential role of itaconate in mitochondrial ROS. Remodelling of the TCA cycle is a metabolic adaptation mechanism that accompanies macrophage activation, in which endogenous metabolites are indispensable in regulating specific processes of the inflammatory response. Our previous research showed that Sirt1 promotes M2Mφ to alleviate crystal‐induced kidney damage, and the transformation of this phenotype is essentially driven by metabolic reprogramming. To further explore the positive role of Sirt1 in macrophages, we extracted BMDMs (Figure S1F) and performed RNA‐seq analysis. Gene Ontology enrichment revealed that the majority of differentially expressed genes were associated with immunity and inflammation regulation (Figure 1G), and Kyoto Encyclopedia of Genes and Genomes pathway analysis identified three predominant signalling pathways: ‘Toll‐like receptor’, ‘NF‐kappa B’ and ‘cell adhesion’ pathways (Figure S1G). Through integrative analysis, we identified 35 coregulated genes implicated in both inflammation and immune regulation (Figure 1H), among which the abundance of Irg‐1 was significantly increased in activated macrophages (Figures 1I and S1H). Interestingly, Irg‐1 synthesises itaconate using cis‐aconitate (a TCA cycle intermediate) as a substrate, indicating that Irg‐1 might be a pivotal target of inflammation and metabolism.

### 3.2 Sirt1 in macrophages protects against CaOx‐induced TECs damage via the Irg‐1/Sdha axis

**FIGURE 2:**
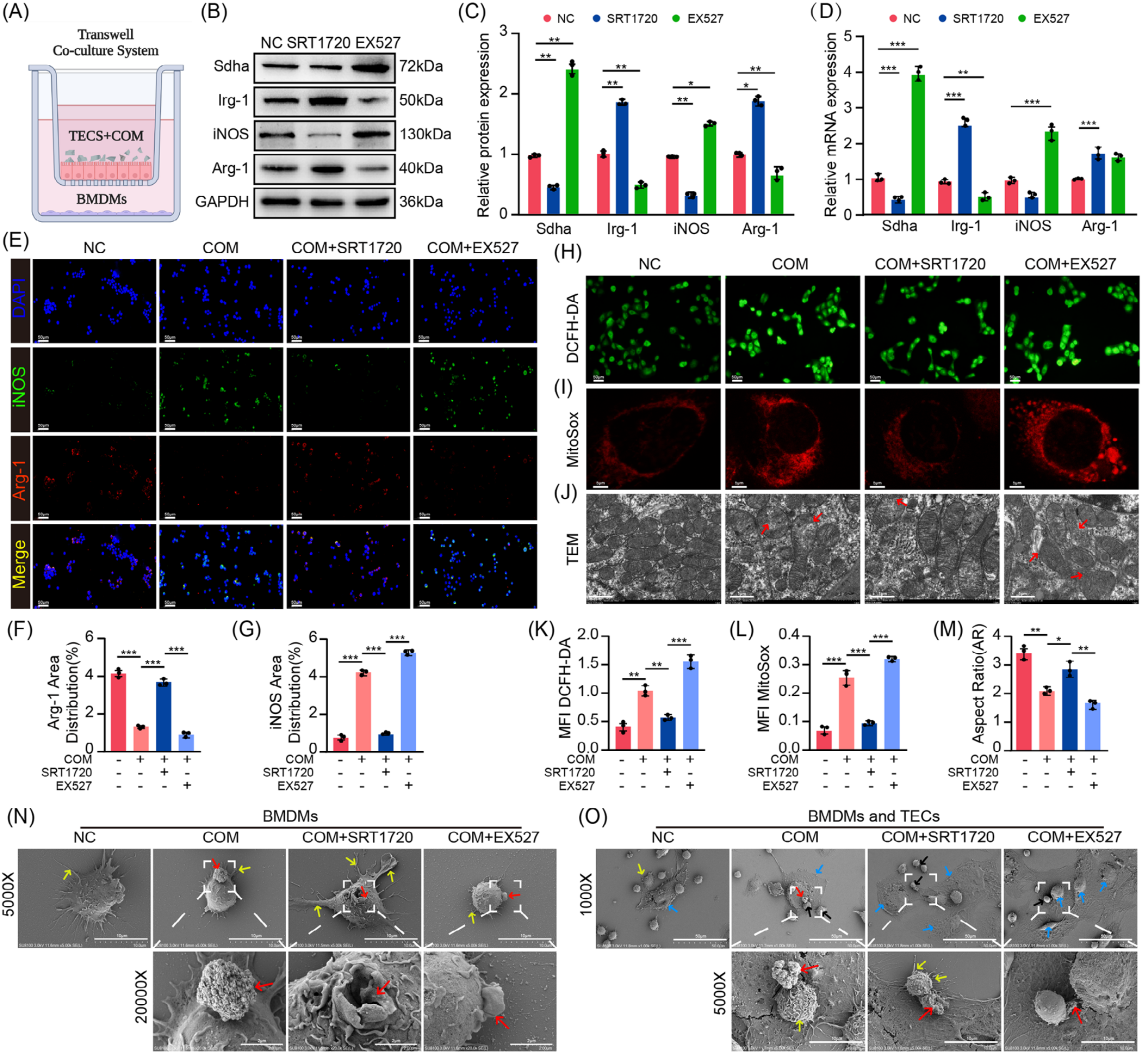
Sirt1 protects against TECs mitochondrial damage and pro‐inflammatory polarisation of macrophages. (A) Coculture schematic of BMDMs and COM‐TECs. (B and C) Western blot and quantification of Irg‐1, Sdha and macrophage polarisation marker levels under COM or Sirt1 activation/inhibition in the coculture system (*n* = 3). (D) Relative mRNA analysis of Irg‐1, Sdha and macrophage polarisation marker levels under COM or Sirt1 activation/inhibition in the coculture system (*n* = 3). (E–G) IF showed the proportion of macrophage iNOS (green) and Arg‐1 (red) (200×, scale bar: 50 µm) (*n* = 3). The cytoplasmic (H and I) (200×, scale bar: 50 µm) and mitochondrial (K and L) (2000×, scale bar: 5 µm) ROS levels of TECs were observed by fluorescence microscopy and laser confocal microscopy (*n* = 3). (J and M) Mitochondrial morphology was analysed by TEM (10 000×, scale bar: 1 µm). Red arrows reveal swollen and disrupted mitochondria. (N) SEM demonstrated the micromorphology of crystals phagocytosed by macrophages (yellow arrow: macrophage pseudopodia; red arrow: COM crystals. Up channel: 5000×, scale bar: 10 µm; down channel: 20 000×, scale bar: 2 µm) (*n* = 3). (O) SEM indicated the morphology of macrophage phagocytic crystals in the coculture system (black arrow: macrophages; blue arrow: TECs; yellow arrow: macrophage pseudopodia; red arrow: COM crystals. Up channel: 1000×, scale bar: 50 µm; down channel: 5000×, scale bar: 10 µm) (*n* = 3). MFI: mean fluorescence intensity. Data are presented as mean ± SD (*n* = 3–6 per group). *p < .05, **p < .01, ***p < .001.

SDH is a complex formed by anchoring subunits (Sdhc and Sdhd) connecting catalytic subunits (Sdha and Sdhb) on the inner mitochondrial membrane (Figure S2A).^28^ To determine which subunits were regulated by Sirt1, qRT‐PCR and Western blotting were performed and found that Sirt1 significantly inhibited Sdha expression (Figure S2B–D). Based on previous studies, we hypothesised that the anti‐inflammatory potential of Sirt1 may be achieved through the Irg‐1/Sdha axis. To investigate the potential mechanism, we constructed a CaOx crystal microenvironment coculture system (Figure 2A). Our results indicated that Sirt1 promoted Irg‐1 expression while limiting the levels of pro‐inflammatory genes, including Sdha, HIF‐1α and IL‐1β (Figures 2B–D and S2E,F). Additionally, the levels of Arg‐1 (an M2Mϕ marker) were increased, while those of iNOS (an M1Mϕ marker) were decreased (Figure 2B–D). Using Immunofluorescence (IF) and flow cytometry, we further demonstrated that Sirt1 improved the polarisation of M2Mϕs. Inhibition of Sirt1 with EX527 resulted in the opposite situation, suggesting that Sirt1 promotes M2Mϕ polarisation and inhibits the expression of downstream inflammatory genes (Figures 2E–G and S2G,H). Excitingly, Sirt1 activation significantly increased the abundance of itaconate (Figure S2I). In other words, Sirt1 increases itaconate by up‐regulating Irg‐1, thereby regulating macrophage metabolism and polarisation conversion.

Subsequently, we assessed the protective effects of Sirt1 on COM‐induced oxidative damage in TECs. Confocal microscopy revealed that Sirt1 reduced both mitochondrial (primary source of ROS) and cytoplasmic ROS in TECs (Figure 2H,I,K,L). Transmission electron microscopy (TEM) scanning showed that COM exposure led to mitochondrial swelling and damage, while Sirt1 protected against oxidative damage and promoted the normalisation of mitochondrial morphology (Figure 2J,M). Furthermore, we observed that mitochondrial membrane potential recovered to a high level in response to Sirt1, as a decreased mitochondrial membrane potential is an early apoptosis indicator (Figure S2J,K). Meanwhile, we found that com leads to a significant decrease in ATP levels and ATPase activity, while Sirt1 can alleviate this effect (Figure S2L,M). To better elucidate the differences in the microstructure and morphology of macrophages, scanning electron microscopy (SEM) was performed on the TEC‐BMDM coculture system. The findings showed that under Sirt1 intervention, BMDMs exhibited larger cellular volume, increased surface microvilli and folds and active pseudopodia (indicative of enhanced phagocytosis) compared with the COM group (Figures 2N and S2N,O). We then used SEM to observe macrophage phagocytosis and encapsulation of crystals exposed to epithelial cells. While the adhesion of COM to the surface of TECs triggered an inflammatory response, macrophages rapidly recruited and extended pseudopodia to engulf crystals, which was exceptionally significant under the regulation of Sirt1 (Figure 2O). In addition, to assess quantitative data of macrophage engulfment rate, we conducted macrophage fluorescence phagocytosis experiments. The results indicated that Sirt1 significantly increased the phagocytic amount of COM crystals in macrophages (Figure S2P).

### 3.3 Conditional knockout of Sirt1 in mouse exacerbates crystal deposition and inflammation

**FIGURE 3:**
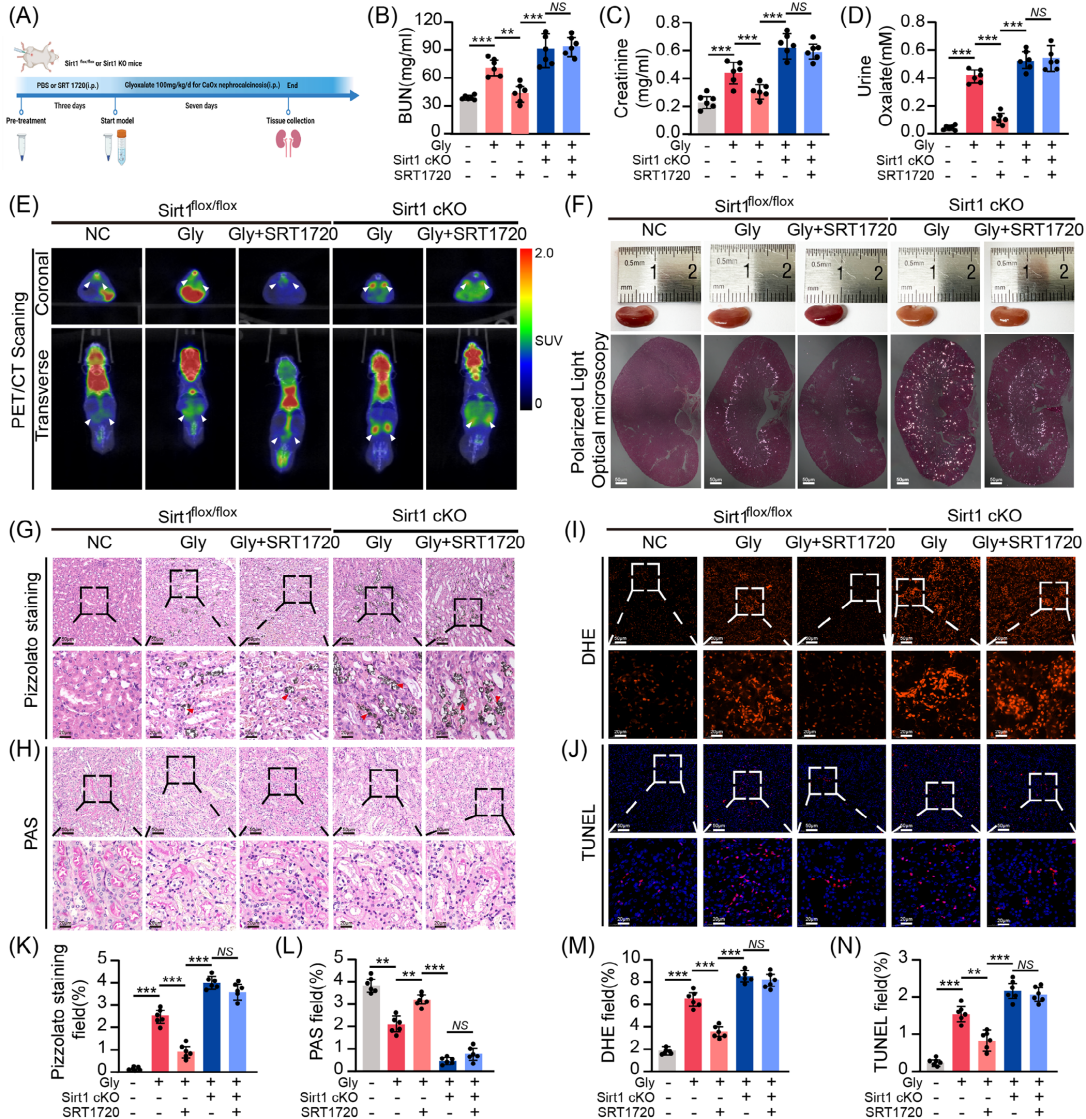
Sirt1 cKO aggravated renal CaOx crystal deposition and oxidative damage. (A) Diagram of the experimental design. (B and C) BUN and creatinine levels in each group of mice. (D) Urine oxalate levels in each group of mice. (E) ^18^F‐FDG PET/CT was used to assess the inflammatory status of the kidneys in Sirt1^flox/flox^ and Sirt1 cKO mice (*n* = 6). (F) Polarised light optical microscopy showed CaOx crystal deposition in different groups (200×, scale bar: 50 µm). (G and K) Pizzolato staining confirmed that CaOx crystal deposition. (H, I and L, M) PAS and DHE staining revealed oxidative damage to TECs (upper channel: 200×, scale bar: 50 µm; lower channel: 600×, scale bar: 20 µm). (J and N) TUNEL staining showed TECs injury and apoptosis (upper channel: 200×, scale bar: 50 µm; lower channel: 600×, scale bar: 20 µm). SUV: standardised uptake value. *p < .05, **p < .01, ***p < .001.

To confirm the potential mechanism of Sirt1 in vivo, we performed a series of experiments on Sirt1 conditional knockout (cKO) and Sirt1^flox/flox^ mice with either PBS or SRT1720 followed by 7 days of induction of the CaOx nephropathy model using Gly (Figure 3A). IF showed that Sirt1 expression was significantly decreased in cKO cells (Figure S3A). We found no significant differences in blood urea nitrogen (BUN), creatinine in mice treated with Gly and Sirt1 agonists simultaneously, while Sirt1 agonists pre‐administered for 3 days effectively protected kidney function and reduced urinary oxalate levels (Figures 3B–D and S3B–D). PET/CT scans revealed an elevated accumulation of ^18^F‐FDG in the kidneys of the Gly group compared with the control, indicating an aggravation of inflammation. Interestingly, SRT1720 markedly diminished ^18^F‐FDG accumulation, thereby substantially mitigating the kidney's inflammatory status. The severity of kidney inflammation appeared to increase following Sirt1 cKO and SRT1720 may not rescue renal injury in Sirt1 cKO group (Figures 3E and S3E). Subsequently, in order to further confirm the source of inflammatory signals, we performed IF staining on macrophage marker (F4/80) and found that macrophages increased after Sirt1 cKO and were positively correlated with inflammatory infiltration, indicating that the validation signal mainly came from macrophage infiltration (Figure S3F). The appearance of the kidney also serves as a valuable indicator in assessing renal injury. Upon administration of Gly, we noticed a whitening of the mouse kidneys, particularly in Sirt1 cKO mice, which suggests intense inflammation. This condition was notably alleviated following Sirt1 activation (Figure 3F). Through polarised light optical microscopy and Pizzolato staining, we demonstrated obvious CaOx crystal deposition in the kidneys of the Sirt1 cKO group. However, this accumulation was significantly reversed through Sirt1 activation in Sirt1^flox/flox^ group (Figures 3F,G,K and S3G). In addition, Immunohistochemistry (IHC) and ELISA analysis have shown that knocking out Sirt1 increases the levels of downstream pro‐inflammatory factors including Sdha and IL‐β (Figure S3H–L). Furthermore, Periodic Acid‐Schiff (PAS) staining, Dihydrorhodamine (DHE) and Terminal Deoxynucleotidyl Transferase‐mediated dUTP Nick End Labeling (TUNEL) assays also affirmed these observations, revealing the beneficial effect of Sirt1 in protecting TECs from oxidative damage induced by CaOx crystals (Figure 3H–J, L–N).

### 3.4 H3K27me3 and H3K4me3 mediate Sirt1 regulation of Irg‐1 and Sdha

**FIGURE 4:**
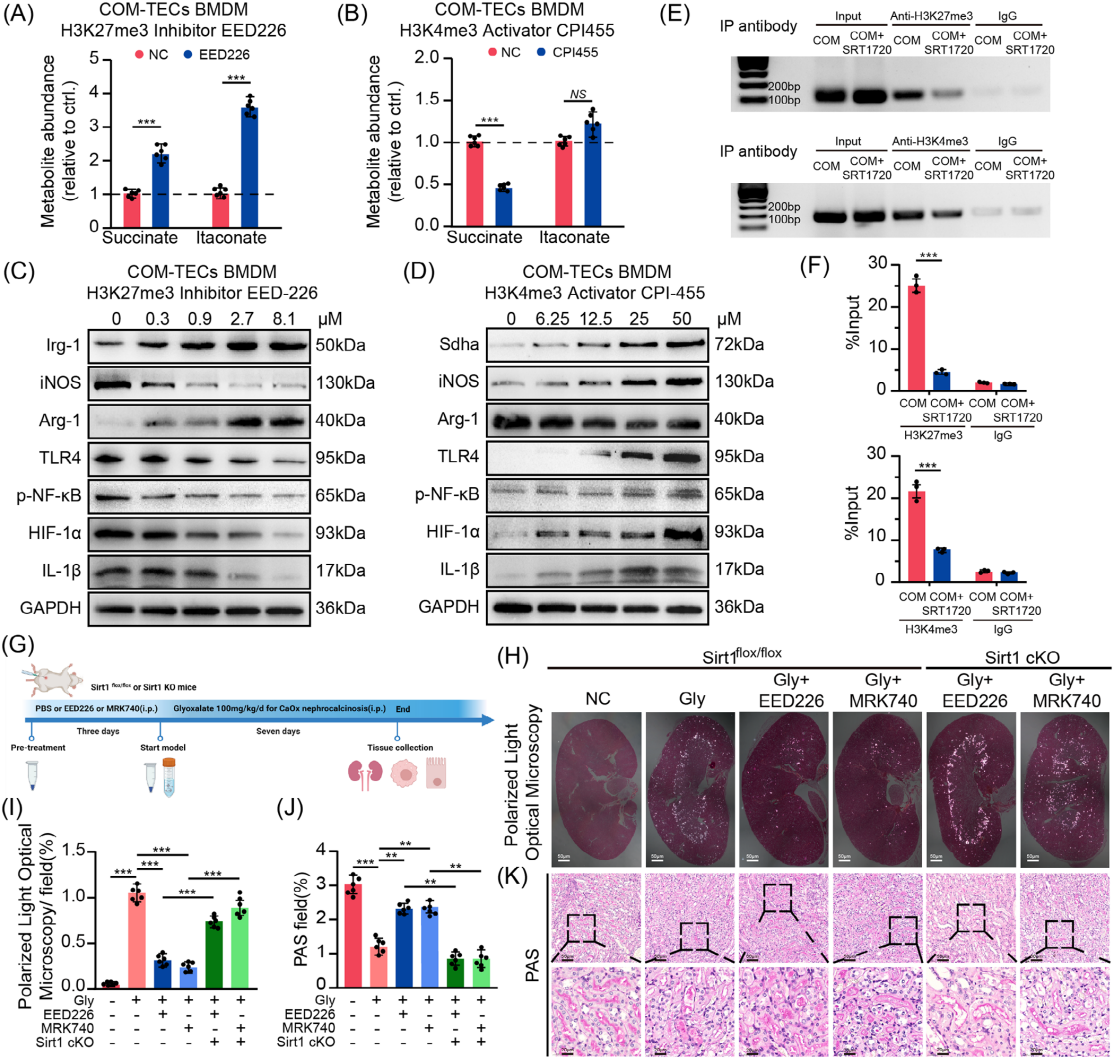
Histone regulation of downstream inflammatory genes and reversion experiments in macrophages. (A) Metabolic abundance of succinate and itaconate under H3K27me3 down‐regulation. (B) Metabolic abundance of succinate and itaconate under H3K4me3 up‐regulation (*n* = 3). (C) Western blot analysis indicated the regulation of macrophage inflammation and polarisation by inhibition of H3K27me3 (*n* = 3). (D) Western blot revealed the regulation of macrophage inflammation and polarisation by activation of H3K4me3 (*n* = 3). (E and F) ChIP and ChIP–qPCR analysis of histone binding to the Sirt1 promoter in BMDMs treated with SRT1720 (*n* = 3). (G) Diagram of the experimental design. (H and I) Pizzolato staining detected CaOx crystal deposition (upper channel: 200×, scale bar: 50 µm; lower channel: 600×, scale bar: 20 µm) (*n* = 6). (J and K) PAS staining revealed oxidative damage to TECs (upper channel: 200×, scale bar: 50 µm; lower channel: 600×, scale bar: 20 µm). *p < .05, **p < .01, ***p < .001.

Previous findings demonstrated that Sirt1 modulates the expression of Irg‐1 and sdha, yet the precise regulatory mechanism remains unclear. To determine whether Sirt1 directly governs the transcriptional activity of these genes, chromatin immunoprecipitation (ChIP) coupled with quantitative PCR (qPCR) was performed to examine Sirt1 occupancy at their promoter regions. Notably, the ChIP–qPCR results revealed no significant enrichment of Sirt1 at the promoter regions of Irg‐1 or sdha, suggesting that Sirt1 does not directly bind to these loci (Figure S4A). These observations imply that Sirt1 likely regulates Irg‐1 and sdha expression through indirect mechanisms, potentially involving epigenetic regulators. As Sirt1 functions as a histone deacetylase, its deacetylates histones, which in tandem with methylation creates more resilient epigenetic marks. Leveraging ChIP‐seq data from the Cistrome Dataset and UCSC Genome Database, we predicted a significant enrichment of H3K27me3 and H3K4me3 within the transcription start sites of Irg‐1 and Sdha, respectively. Sirt1, by deacetylating EZH2, diminishes its capacity to methylate H3K27 and H3K4. Consequently, this attenuates the inhibitory function of H3K27me3 and the transcription‐enhancing role of H3K4me3 on target genes.^29^ This process was also validated in our preliminary experiments (Figure S4B,C). Consequently, it is plausible that Sirt1 controls the metabolism of inflammatory molecules and organic acids via H3K27me3 and H3K4me3, thereby curbing the onset and progression of CaOx nephropathy. Consistent with this, our findings demonstrated that as H3K27me3 levels decreased, there was a significant up‐regulation of itaconate and Irg‐1. In contrast, the expression of pro‐inflammatory genes, such as HIF‐1α and IL‐1β, displayed a negative correlation (Figures 4A,C and S4D). In addition, enhanced Irg‐1 expression fostered the polarisation of macrophages towards M2Mφs (Arg‐1) while concurrently reducing M1Mφs (iNOS) (Figures 4A,C and S4D), indicating that inhibiting H3K27me3 fosters the phagocytosis ability of macrophages. Conversely, the up‐regulation of H3K4me3 led to a contrasting outcome: H3K4me3 significantly elevated Sdha expression, fostering the expression of inflammatory genes, while the abundance of succinate markedly declined (Figures 4B,D and S4E).

Additionally, a ChIP experiment was conducted to demonstrate the binding of H3K27me3 and H3K4me3 to Irg‐1 and Sdha, respectively. Our findings indicated a significant reduction in H3K27me3 binding to the promoter region of Irg‐1 following Sirt1 activation (Figure 4E,F), while the binding of H3K4me3 to Sdha was concurrently inhibited (Figure 4E,F). Consequently, our in vitro experiments suggested that Sirt1 may modulate the expression levels of Irg‐1 and Sdha by altering histone enrichment in their promoter regions, thereby alleviating renal inflammatory damage and the progression of CaOx nephropathy.

### 3.5 Suppression of histones partially reverses the effects of Sirt1 knockdown on renal crystal deposition and the inflammatory response

To further identify Sirt1 in protecting against CaOx crystal‐induced kidney injury via histones in vivo, we treated both flox/flox and Sirt1 cKO mice with inhibitors of H3K27me3 and H3K4me3 (Figure 4G). Polarised light optical microscopy revealed that EED226 and MRK740 significantly reduced the deposition of kidney crystals in Sirt1^flox/flox^ mice compared with the control group, while this effect was not evident in the Sirt1 cKO mice (Figure 4H,I). Moreover, PAS, DHE and TUNEL staining indicated that renal oxidative damage was notably mitigated after H3K27me3 and H3K4me3 down‐regulation but reappeared following Sirt1 cKO (Figures 4J,K and S4F–I).

Furthermore, our results indicated that inhibiting both histones significantly diminishing the levels of Sdha and IL‐1β. In contrast, when Sirt1 was knocked out, the opposite results were observed (Figure S5A–E). These findings suggest that Sirt1 governs the metabolism and inflammatory response of macrophages through histones (Figure 7).

### 3.6 Sirt1 mitigates CaOx‐induced renal inflammatory and oxidative injury via H3K27me3/Irg‐1–H3K4me3/Sdha regulation

**FIGURE 5:**
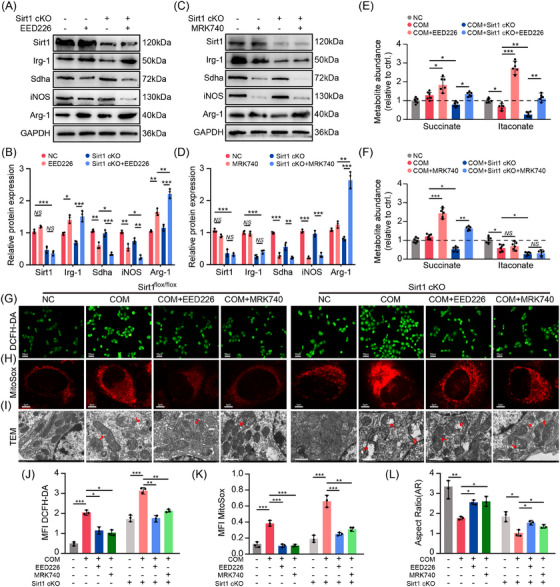
Sirt1 alleviates renal inflammation and oxidative damage caused by CaOx crystallisation in mice through the H3K27me3/Irg‐1–H3K4me3/Sdha pathway. (A) Metabolic abundance of succinate and itaconate under Sirt1 cKO and H3K27me3 down‐regulation (*n* = 3). (B) Metabolic abundance of succinate and itaconate under Sirt1 cKO and H3K4me3 down‐regulation. (C and D) Inhibition of Sirt1 concomitantly down‐regulated H3K27me3 on the protein levels of Irg‐1 and downstream inflammatory genes analysed by Western blot (*n* = 3). (E and F) Inhibition of Sirt1 concomitantly down‐regulated H3K4me3 on the protein levels of Sdha and downstream inflammatory genes analysed by Western blot (*n* = 3). The cytoplasmic (G and J) (200×, scale bar: 50 µm) and mitochondrial (H and K) ROS levels of TECs were observed by fluorescence microscopy and laser confocal microscopy (*n* = 3). (I and L) Mitochondrial morphology was analysed by TEM (10 000×, scale bar: 1 µm) (*n* = 3). Red arrows reveal swollen and disrupted mitochondria. MFI: mean fluorescence intensity. Data are presented as mean ± SD (*n* = 3 per group). *p < .05, **p < .01, ***p < .001.

To assess the validity of this pathway in vitro, we extracted BMDMs from Sirt1 cKO mice and cocultured them with COM‐induced TECs. Our results suggested that the abundance of Irg‐1 and itaconate declined following Sirt1 cKO in BMDMs, in contrast to the increased levels of Sdha and pro‐inflammatory genes (Figure 5A,B,E). Depression of H3K27me3 triggered an up‐regulation of Irg‐1, which corresponded to a decline in inflammatory gene expression and a notable augmentation in itaconate levels. Interestingly, by simultaneously inhibiting H3K27me3 on the basis of Sirt1cko, we observed an increase in Irg‐1 levels and inhibition of the inflammatory pathway. This further indicates that Sirt1 regulates the expression of Irg‐1 and inflammatory response through H3K27me3 (Figures 5A,B,E and S6A,B). In addition, the down‐regulation of H3K4me3 markedly impeded Sdha and enhanced succinate levels, while Irg‐1 levels remained stable (Figure 5C,D,F). We observed a decreased inflammatory response following the concurrent inhibition of Sirt1 and H3K4me3. This is attributable to the blockade of pro‐inflammatory signal transduction triggered by Sirt1 down‐regulation when H3K4me3 was simultaneously inhibited (Figures 5C,D,F and S6C,D). Data from flow cytometry revealed that down‐regulating H3K27me3 and H3K4me3 notably bolstered M2 polarisation while attenuating M1 polarisation. Interestingly, the suppression of Sirt1 yielded contrasting results (Figure S6E,F). Furthermore, we assessed the degree of mitochondrial oxidative damage in TECs. Our findings, based on DCFH‐DA, MitoSox and TEM, revealed that inhibition of Sirt1 amplified COM‐induced ROS production. In contrast, histone down‐regulation helped mitigate this damage. When both Sirt1 and histones were inhibited, we observed a recovery of mitochondrial damage. However, the severity remained higher than that in Sirt1 cKO mice (Figure 5G–I,J–L). Mitochondrial membrane potential detection and ATP levels also well demonstrated the protective effect of Sirt1 (Figure S6G–J). This underscores that Sirt1 deficiency plays a pivotal role in the progression of mitochondrial oxidative damage.

### 3.7 Effective relief of kidney crystallisation and inflammatory damage through exogenous itaconate supplementation

**FIGURE 6:**
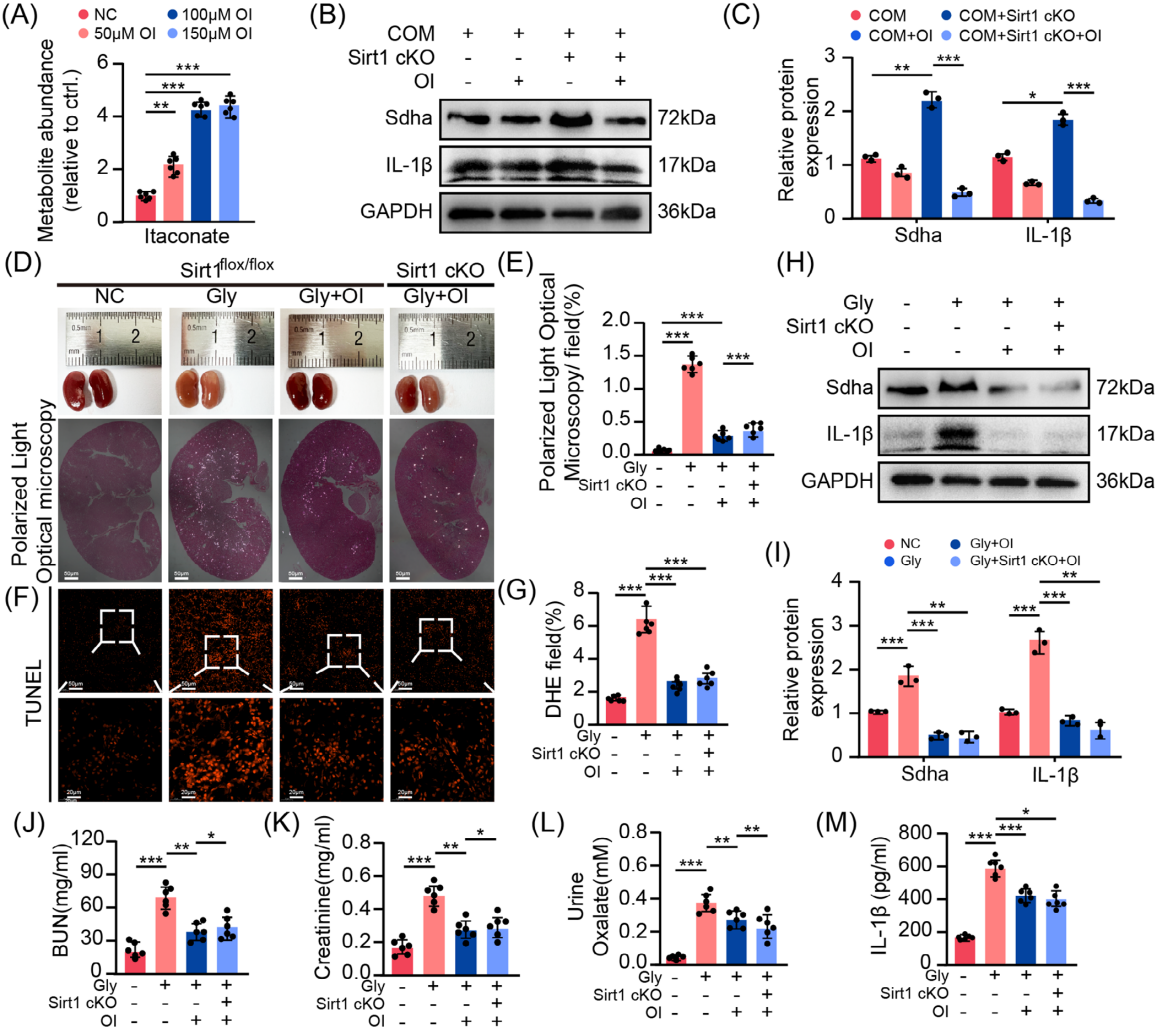
4‐Octyl itaconate (OI) effectively relieves kidney crystallization and inflammatory damage. (A) Metabolic abundance of itaconate under OI treatment in BMDMs (*n* = 3). (B and C) Western blot showing the Sdha level under OI treatment in Sirt1 cKO BMDMs (*n* = 3). (D and E) Kidney colour and polarised light optical microscopy detection of Sirt1^flox/flox^ and Sirt1 cKO mice treated with OI (*n* = 6). (F and G) TUNEL staining showed TECs injury and apoptosis (upper channel: 200×, scale bar: 50 µm; lower channel: 600×, scale bar: 20 µm) (*n* = 6). (H and I) Western blot showing the Sdha level under OI treatment in kidney tissue (*n* = 3). (J and K) BUN and creatinine levels in each group of mice. (L) Urine oxalate levels in each group of mice (*n* = 6). (M) IL‐1β levels measured by ELISA in cell culture supernatants from mice (*n* = 6). *p < .05, **p < .01, ***p < .001.

To ascertain the anti‐crystallisation and anti‐inflammatory potential of itaconate, we administered OI, a cell‐permeable itaconate surrogate, to BMDMs.^16^, ^27^ The hydrolysis process of OI increased the intracellular itaconate level (Figure 6A). As the concentration of OI increased, there was a notable decrease in Sdha and IL‐1β levels (Figure S7A,B). Notably, in Sirt1 cKO BMDMs, the abundance of Sdha was significantly diminished by OI supplementation (Figure 6B,C). In a subsequent in vivo experiment, we found that the kidneys of mice treated with OI resembled those of the control group, in stark contrast to the Gly group (Figure 6D). There was a significant reduction in crystal deposition and apoptotic damage within the OI‐treated group (Figure 6D–G), and OI administration significantly reduced oxalate levels and improved renal function in mice (Figure 6J–L). Moreover, similar to the pattern observed in BMDMs, OI supplementation led to a notable decrease in Sdha and IL‐1β expression in kidney tissue (Figures 6H,I,M and S7C–F). This underlines the valuable role of exogenous itaconate's inhibitory effect on Sdha in mitigating crystal‐induced renal injury.

**FIGURE 7:**
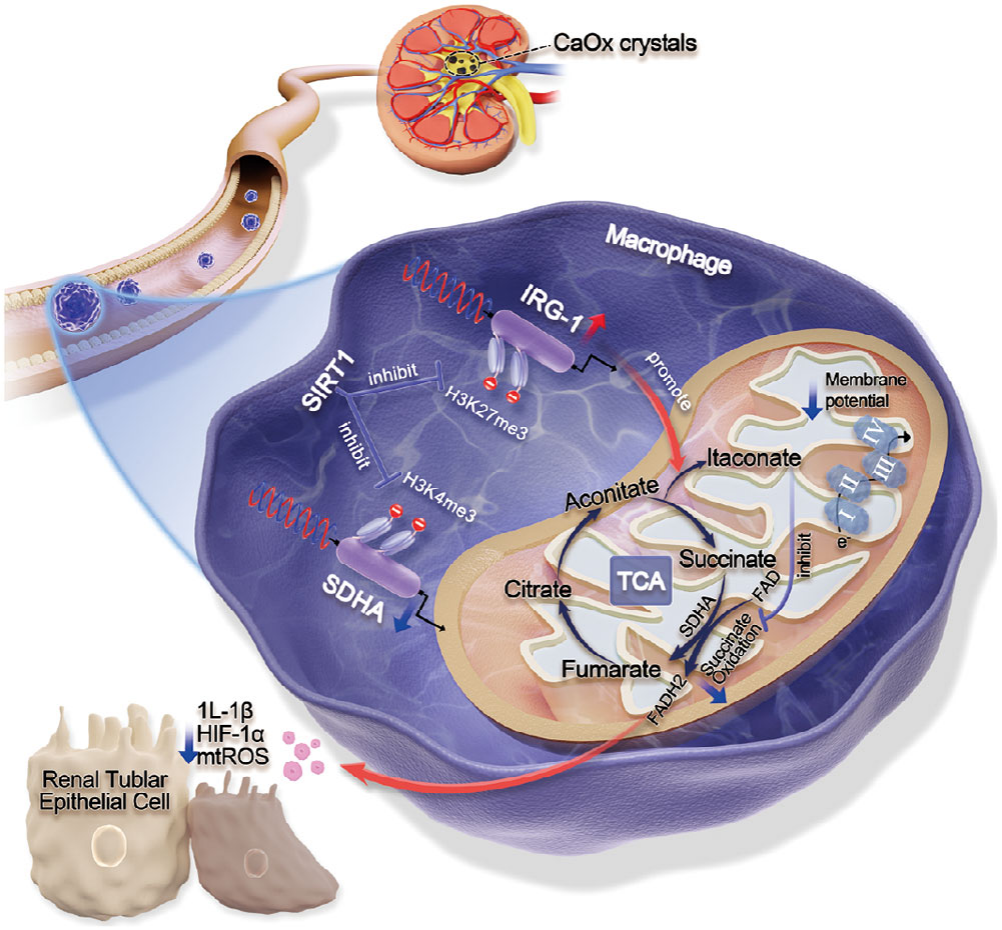
Schematic diagram for therapeutic effects of Sirt1 on CaOx induced renal inflammatory and oxidative injury via the H3K27me3/Irg‐1–H3K4me3/Sdha pathway.

## 4 DISCUSSION

Specific metabolic processes drive immune cell activation, with the TCA cycle emerging as the central hub of immune metabolism for macrophages. Serving as the final metabolic pathway of three key nutrients, the TCA cycle accumulates crucial intermediates, such as succinate, itaconate and α ketoglutarate, which activate immune cells with its unique signals.^30^ Previous studies have focused on the variations in SDH without elaborating the specific subunit. In COM‐induced macrophages, we observed a surge in Sdha abundance, which stabilises the pro‐inflammatory effects of HIF‐1α and stabilises the release of IL‐1β and mitochondrial ROS via succinate oxidation. Sirt1, through Irg‐1, promotes itaconate levels, which competitively inhibit succinate oxidation mediated by SDH, thereby reducing the inflammatory and oxidative damage inflicted by M1Mϕs. Consistently, constraining SDH with dimethyl malonic acid effectively curbed both ROS production and IL‐1β secretion, hinting at the potential of SDH oxidation limitation as a viable strategy to suppress the release of inflammatory molecules.^31^ Notably, this phenomenon was corroborated in both COM‐induced macrophages and CaOx nephropathy mouse models. Post OI administration, we observed a significant surge in itaconate abundance and a decrease in Sdha expression. Additionally, there was a marked reduction in inflammatory cytokines (IL‐1β) and renal crystal deposition.^32^, ^33^ Moreover, our results indicated that Sirt1 expedited succinate accumulation and reduced fumarate levels, as Sdha inhibition hindered succinate oxidation. However, the levels of ROS and inflammatory cytokines did not exhibit a significant increase, aligning with Lampropoulou et al.’s assertion that succinate oxidation, rather than accumulation, triggers the inflammatory response. It is plausible that other molecules, such as succinate receptor 1 (SUCNR1), could also contribute to the pro‐inflammatory effects of succinate.^34^, ^35^ Further investigation is warranted to determine whether SDH restriction and increased succinate accumulation amplify the pro‐inflammatory effect of SUCNR1. In addition, compared with the anti‐inflammatory effect of itaconate^16^, ^36^ Sirt1 has a more extensive effect. In addition to its powerful anti‐inflammatory and antioxidant effects, Sirt1 also has significant anti‐aging and regulating cellular energy metabolism functions^18^, ^37^, ^38^, ^39^, ^40^ Developing suitable clinical drugs for Sirt1 is highly valuable, as it may be widely effective for multiple diseases and has great clinical significance for promotion.

Furthermore, the polarisation of macrophages plays a pivotal role in the formation and development of CaOx nephropathy.^41^ Song et al.^42^ showed that Sirt1 promotes macrophage M2 polarisation by inhibiting the NOTCH signalling pathway, thereby reducing CaOx crystal deposition in the kidneys. Kletzmayr et al.^43^ identified CaOx crystal inhibitors to alleviate the progression of oxalate nephropathy. Zhu et al.^44^ elucidated the underlying mechanism of androgen receptor regulation of M2 polarisation and inhibition of renal CaOx crystal deposition. Utilising a BMDM‐TEC coculture system, we demonstrated that in vitro activation of Sirt1 mitigates M1Mϕ polarisation while promoting M2Mϕ polarisation, thereby reducing CaOx‐induced inflammatory injury. In addition, we observed the morphological transformation of macrophages into M2Mϕs after Sirt1 activation. Consequently, we propose that Sirt1's regulatory role in macrophage polarisation and inflammation is intricately linked to the formation and progression of CaOx nephropathy, with polarisation serving as an indicative representation of metabolic transformation within macrophages

Research has shown that changes in urinary oxalate concentration can affect the risk of stone formation, and this process is mainly achieved through inflammatory reactions mediated by inflammatory molecules such as NLRP3.^45^, ^46^ Exposure of TECs to CaOx crystals results in excessive free radical production and heightened oxidative stress, ultimately leads to cell death and crystal deposition in the tubulointerstitium,^47^, ^48^ which was also validated in our previous study.^49^ Additionally, the mitochondrial respiratory chain experiences a high level of succinate oxidation. During this process, the mitochondrial respiratory chain oxidises high levels of succinate, reversing electron flow and triggering a phenomenon known as reverse electron transport in complex I, resulting in excessive ROS production.^13^ The protective role of Sirt1 in mitigating ROS‐induced damage is well documented across multiple pathophysiological processes.^50^, ^51^, ^52^ Ye et al.,^53^ for instance, demonstrated how theaflavin, operating through the miR‐128‐3p/Sirt1 axis, aids the antioxidant defense system, thereby reducing crystal‐induced kidney damage. Moreover, augmenting Sirt1 expression with resveratrol significantly curtails crystal deposition and cell damage.^54^ However, the exact mechanism through which Sirt1 curbs inflammation remains elusive. In fact, Sirt1 plays a positive role in various kidney diseases. Whether in diabetes nephropathy or other causes of kidney damage, Sirt1 has shown a strong role in inhibiting inflammation and protecting kidney damage^55^, ^56^, ^57^ However, the mechanism by which Sirt1 reduces kidney damage may be completely the same in different diseases. Our study provides intriguing evidence that the use of Sirt1 to limit succinate oxidation can ameliorate mitochondrial damage, decrease ROS levels, and ultimately minimise CaOx‐induced TECs damage.

Contrasting the dynamic alterations seen in histone acetylation, which typically activate transcription, histone methylation presents as a more stable and versatile epigenetic marker, which is related to gene activation and inhibition according to the characteristics and location of the modification.^58^ ChIP‐seq analysis Studies have reported that H3K9me3 and H3K27me3 play roles in transcriptional inhibition within inactive chromatin.^59^ ChIP‐seq analysis of CpG sites labelled with either hypermethylation or hypomethylation reveals that the enrichment of hypermethylated H3K4me3 notably decreases with the activation of Sirt1.^19^ Moreover, H3K27me3 and H3K4me3 bind to the promoters of inflammatory genes, modulating their expression.^60^, ^61^ Our results indicated that these histone modifications directly bind potential sequence sites in the promoter regions of Irg‐1 and Sdha based on Sirt1 activation. Subsequent in vitro and in vivo experiments support that Sirt1 curbs the release of inflammatory cytokines through H3K27me3 and H3K4me3 histone modifications. These insights suggest a potential role for Sirt1 in interacting with histone modifications in the epigenetic inheritance of CaOx nephropathy, thereby facilitating diverse biological functions. It is worth noting that inhibiting H3K27me3 and H3K4me3 significantly reduces inflammation and renal crystal deposition in TECs, indicating that histone modifications have independent effects. However, their effects may be regulated by upstream molecules such as Sirt1^19^, ^29^ Because histones control the degree of chromatin opening, any changes in their expression can lead to transcriptional changes in genes. Therefore, small molecule drugs that directly target histones also have certain application prospects.

In conclusion, we have uncovered a potential mechanism whereby Sirt1 modulates organic acid metabolism, thus inhibiting CaOx crystallisation progression and mitigating its associated inflammatory and oxidative damage to the kidney. These findings broaden our understanding of Sirt1's physiological roles and illuminate a novel pathway for the regulation of CaOx nephropathy development. Most importantly, Sirt1 agonists may be used as preventative and therapeutic agents for CaOx nephropathy.

## AUTHOR CONTRIBUTIONS

This work was performed in collaboration among all authors. X. Y. Y. and H. R. L. designed the study. X. Y. Y., H. R. L. and C. D. carried out the experiments. Y. J. Z., X. L. W. and B. L. performed data analysis. X. Y. Y., Y. G. and T. Z. L. contributed to figures and tables. X. Y. Y. and H. R. L. drafted the manuscript. S. L., X. H. W. and H. X. revised the manuscript. All authors have approved the final manuscript.

## CONFLICT OF INTEREST STATEMENT

The authors declare no conflicts of interest.

## ETHICS STATEMENT

All experiments were approved by the Ethics Committee of Zhongnan Hospital of Wuhan University (MRI2023‐LACA18) and adhered to the NIH Guide for the Care and Use of Laboratory Animals.

## Supporting information



Supporting Information

## Data Availability

All data required to evaluate the conclusions of this manuscript are presented in the paper or the Supplementary Materials. Any data associated with this study are available from the corresponding author upon reasonable request. The RNA‐seq data in this study can be accessed at the Gene Expression Omnibus (https://www.ncbi.nlm.nih.gov/gds/) with accession number GSE192593.
