# Supplementary material for: Sirtuin1 mitigation of calcium oxalate nephropathy via enhancing itaconate abundance through reduction of histone trimethylation
Source: Clin Transl Med. 2025 Aug 18;15(8):e70450. doi: 10.1002/ctm2.70450 (PMC12360365; doi:10.1002/ctm2.70450)
Supplement: Supplementary file 1 — Supporting Information [file CTM2-15-e70450-s001.docx]

**Supplementary Materials**

**Supplementary methods**

**Fig. S1 Expression of Sirt1 in tissues and identification of differentially expressed genes.**

**Fig. S2 Regulation of Sirt1 on downstream inflammatory genes.**

**Fig. S3 Protective effect of Sirt1 on renal crystal deposition.**

**Fig. S4 Regulation of Sirt1 at the histone level.**

**Fig. S5 Protective effect of Sirt1 on renal crystal deposition via H3K27me3 and H3K4me3.**

**Fig. S6 Reversion experiments in macrophage polarization and TECs damage.**

**Fig. S7 OI reduces crystal deposition and Sdha levels.**

**Supplementary Table S1. Plasma BUN, creatinine and urine oxalate in mice.**

**Supplementary Table S2. Primer sequences used for the real-time qPCR analysis.**

**Supplementary Table S3. Antibodies used in the manuscript.**

**Supplementary methods**

**Chemicals and reagents**

SRT1720 (Sirt1 agonist), EX527 (Sirt1 inhibitor), MRK740 (H3K4me3 inhibitor) and 4-octyl itaconate (OI) were purchased from MedChemExpress (USA). CPI455 (H3K4me3 agonist) and EED226 (H3K27me3 inhibitor) were obtained from Selleck (USA). Calcium oxalate monohydrate (COM) were obtained from Sigma (USA, C0350000).

**Renal CaOx crystal detection**

The fixed kidney tissue samples were sectioned and stained with hematoxylin-eosin (HE). The renal crystal deposits were visualized by Pizzolatto staining polarized light optical microphotography (Zeiss, Oberkochen, Germany). ImageJ software was used to quantify CaOx crystals in kidney sections. Assessment of Tubular Injury and Cell Apoptosis: Periodic acid-Schiff (PAS) stain were used to assess renal tubular damage, including tubular atrophy and expansion, epithelial cell apoptosis, intraluminal cast formation, and brush edge loss.^1^ Ten nonrepetitive microscope fields (200×) were randomly selected to calculate the percentage of renal tubular cell damage. Terminal deoxynucleotidyl transferase dUTP nick end labeling (TUNEL) staining was performed using a commercial kit (Beyotime, C1086), and positive cells in the analysis were counted to evaluate renal cell apoptosis.^2^

**Immunohistochemistry (IHC)**

The fixed mouse kidney tissues were sectioned and dewaxed with the mixture of xylene and absolute ethanol. Sections underwent antigen retrieval by microwave heating. After blocking with 5% BSA for 1 h, sections were incubated at 4°C overnight with primary antibodies, followed by evaluation using an Envision HRP Polymer system (Boster, China) and a Leica SCN400 scanner (Leica Biosystems, Germany). The relative expression of each protein was analyzed by ImageJ software.

**PET/CT Imaging**

Positron emission tomography/computed tomography (PET/CT) is a nuclear functional imaging technique used to assess glucose metabolic processes in the body. ^18^F-Fluorodeoxyglucose (^18^F-FDG) is an analog of deoxy glucose that accumulates in large amounts in tissues with high glucose uptake.^3^ It reflects active cellular metabolism and consequently provides an image that portrays glucose utilization. High uptake of ^18^F-FDG is also observed in activated inflammatory cells due to active metabolism and increased glucose consumption.^4^

All mice fasted for at least 12 hours before scanning and were then injected with 200±10 μCi ^18^F-FDG through the tail vein. The images were acquired using the static scan mode (10 minutes) of the Trans-PET BioCaliburn 700 system (Leikan Technology, China). Subsequently, AMIDE software (Massachusetts Free Software Foundation) was used to analyze the standardized uptake-value (SUV) measurements.

**Organic Metabolite Analysis by GC‒MS**

After the treatment, mouse kidney tissue of the same size was taken from each group and washed with cold deionized water. Extracts were dried by vacuum centrifugation, resuspended in 10 mg/mL methoxyamine hydrochloride containing pyridine, and derived with N-(tert-butyldimethylsilyl)-N-methyltrifluoroacetamid. Metabolite abundances are expressed relative to an internal standard (D-myristic acid).^5^

**Measurement of BUN, creatinine and oxalate**

After the mice were anesthetized, the plasma of mice in each group was collected. After standing at room temperature for 1h, centrifuge at 2000rpm for 15 minutes. Subsequently, serum BUN and creatinine levels were measured using commercial kits (Stanbio Laboratory, USA). After the mice were sacrificed, the abdominal cavity was exposed, and the urine was collected. After thawing, the oxalate concentration in urine samples was assessed using an oxalate assay kit (Sigma-Aldrich, MAK315). The values of plasma BUN, creatinine and urine oxalate in mice are shown in Table S1.

**Enzyme-linked immunosorbent assay (ELISA)**

Secreted interleukin-1β (IL-1β) levels in cell culture supernatants were quantified using commercially available ELISA kits (DY401, R&D Systems) according to the manufacturer's protocol. Briefly, supernatants were collected and centrifuged at 4°C to remove cellular debris. Then, samples were prepared in assay diluent and loaded onto antibody-coated 96-well plates. Colorimetric detection was performed using a SpectraMax i3x microplate reader (Molecular Devicesmd). Cytokine concentrations were calculated against a standard curve.

**Quantitative real-time PCR**

Total RNA was extracted from kidney tissue and cultured cells with TRIzol reagent (Invitrogen, USA) and then reverse transcribed into cDNA using a PrimeScript RT reagent kit (Vazyme, China). SYBR Green Master Mix (Vazyme, China) was used to perform qRT‒PCR based on standard protocols. All primers used in the experiment are shown in Table S2.

**Chromatin immunoprecipitation (ChIP) assay**

To further verify the binding of histones at predicted sites of downstream genes, ChIP experiments were performed using a ChIP kit (Millipore). The BMDMs activated by Sirt1 were fixed, cross-linked, and fragmented by ultrasound and then coimmunoprecipitated with Sirt1-, H3K27me3- and H3K4me3-specific antibodies, uncross-linked, and DNA fragments were recovered. Finally, primers were designed according to the predicted sites to amplify the corresponding binding regions, and parallel qPCR was used to detect the transcriptional regulation ability of H3K27me3 and H3K4me3 on Irg-1 and Sdha promoter binding sites after Sirt1 regulation.

**Western Blot (WB)**

RIPA lysis buffer (Servicebio) containing protease inhibitors was used to extract total protein. After detecting the protein concentration using the BCA protein assay kit (Servicebio), the tissue extract was electrophoresed by 10% SDS-polyacrylamide gel and transferred to PVDF membranes. Then, the membranes were incubated with primary antibodies overnight at 4°C. After incubation with HRP-labeled secondary antibody at 26°C for 1-2 hours, the membranes were visualized with LumiBlue (TM) ECL Express (Novus). ImageJ software was used for qualification. All experiments were replicated independently three times. Antibodies used in the manuscript are shown in Table S3.

**Flow cytometry**

To analyze the polarization of macrophages, cocultured BMDMs were separated by F4/80 and CD11b (BD Biosciences, USA) flow labeling. On this basis, CD86 and CD206 antibodies (BD Biosciences, USA) were used to distinguish M1 and M2 macrophage. In addition, to assess the mitochondrial membrane potential, cells were obtained and stained according to the instructions of the JC-1 kit (G1515; Servicebio). The fluorescence intensity of stained cells was analyzed by flow cytometry (CYTOFLEX, Beckman).

**Immunofluorescence**

BMDMs were plated on glass slides and fixed with 4% paraformaldehyde. Triton X-100 was used to permeabilizing cells, and blocked with 3% BSA. Then, cells were incubated with primary antibodies against Arg-1 (Abclone, A1847) and iNOS (Proteintech, 80517-1-RR)  in blocking buffer at 4°C for 16 h. Subsequently, simples were incubated with fluorescent antibodies against CY3 (Servicebio, GB21303) or FITC (Servicebio, GB22303) for 2 hours in the dark. Finally, counterstain the nucleus with DAPI. All images were captured with a fluorescence microscope (OLYMPUS IX71, Japan).

**Detection of ROS**

The treated TECs were incubated with 10 µmol/ml DCFH-DA (S0033, Beyotime Biotech, China) at 37°C in the dark for 20 minutes. In addition, TECs were incubated with 100 nmol/ml MitoSOX Red ([HY-D1055](https://www.medchemexpress.cn/mitosox-red.html), MCE) at 37°C for 15 minutes, the cells were washed with 37°C prewarmed PBS. The mouse kidney tissues were embedded in optimum cutting temperature (O.C.T.) and cut into thin slices by a cryostat (Leica CM1950, Germany). Frozen kidney sections were incubated with dihydroethidium (DHE) at 37°C for 30 minutes. All images were observed under a fluorescence microscope (OLYMPUS IX71, Japan). ImageJ software was used to quantify the level of ROS.

**Observation of Mitochondrial Microstructure**

After the mice were sacrificed, the kidney was immediately fixed in 2.5% glutaraldehyde. The glutaraldehyde was removed with PBS, and ultrathin sections were prepared and fixed in 1% osmium tetroxide solution for 1 hour. A transmission electron microscope (Hitachi, Japan) was then used to observe ultrathin sections. The shape transition of mitochondria was quantified by the aspect ratio (AR), and at least 3 mitochondria were selected for AR calculation in each field of view.

**Evaluation of macrophage phagocytosis by scanning electron microscopy (SEM)**

BMDMs or TECs inoculated onto glass slides were fixed with 2.5% glutaraldehyde for 15 min. The samples were dehydrated with ethanol and coated with conformal gold palladium. Then, a Hitachi cold field emission SEM (regulus8100, Japan) was performed to acquire the images.

**ATP detection**

After collecting cells, lyse them in cryolysis buffer. ATP quantification was detected using a commercial chemiluminescence assay kit (Beyotime, S0026). Briefly, 100 μL of testing solution was added to each well and pre-incubated at 25°C for 5 min to degrade background ATP. Subsequently, add 20 μL of cell lysate or ATP standard (0.1-10 μM). Relative light unit (RLU) values were immediately quantified using a GloMax® luminometer (Promega).

**ATP synthase detection**

Following cell collection, samples were lysed in ice-cold lysis buffer supplemented with protease inhibitor cocktail to preserve enzymatic integrity. Subsequently, the detection working solution was added in strict accordance with the manufacturer's protocol (Elabscience, E-BC-K838-M). Optical density (OD) values at 340 nm were quantified using 96-well plates. ATP synthase activity was calculated through the kinetic equation provided in the assay kit documentation.

**Fluorescent labeling of COM crystals and macrophage phagocytosis assay**

COM crystals were co-incubated with a FITC-conjugated anti-rabbit probes under light-protected conditions for 4-6h. Following incubation, the labeled crystals were subjected to three sequential PBS washes. Then, BMDMs were directly cultured with labeled crystals for 4-6h, and incubated with the cytoskeletal protein (f-actin) and nuclear staining (DAPI). Subsequently, cellular internalization was quantified through fluorescence microscopy imaging. The fluorescence intensity in macrophages reflects the amount of crystals engulfed.

**Supplementary references**

1. He C, Imai M, Song H, Quigg RJ, Tomlinson S. Complement inhibitors targeted to the proximal tubule prevent injury in experimental nephrotic syndrome and demonstrate a key role for C5b-9. *J Immunol*. May 1 2005;174(9):5750-7. doi:10.4049/jimmunol.174.9.5750

2. Mulay SR, Desai J, Kumar SV, et al. Cytotoxicity of crystals involves RIPK3-MLKL-mediated necroptosis. *Nat Commun*. Jan 28 2016;7:10274. doi:10.1038/ncomms10274

3. Cherry SR, Badawi RD, Karp JS, Moses WW, Price P, Jones T. Total-body imaging: Transforming the role of positron emission tomography. *Sci Transl Med*. Mar 15 2017;9(381)doi:10.1126/scitranslmed.aaf6169

4. Bleeker-Rovers CP, de Sévaux RG, van Hamersvelt HW, Corstens FH, Oyen WJ. Diagnosis of renal and hepatic cyst infections by 18-F-fluorodeoxyglucose positron emission tomography in autosomal dominant polycystic kidney disease. *Am J Kidney Dis*. Jun 2003;41(6):E18-21. doi:10.1016/s0272-6386(03)00368-8

5. Vincent EE, Sergushichev A, Griss T, et al. Mitochondrial Phosphoenolpyruvate Carboxykinase Regulates Metabolic Adaptation and Enables Glucose-Independent Tumor Growth. *Mol Cell*. Oct 15 2015;60(2):195-207. doi:10.1016/j.molcel.2015.08.013

**Supplemental Figures**


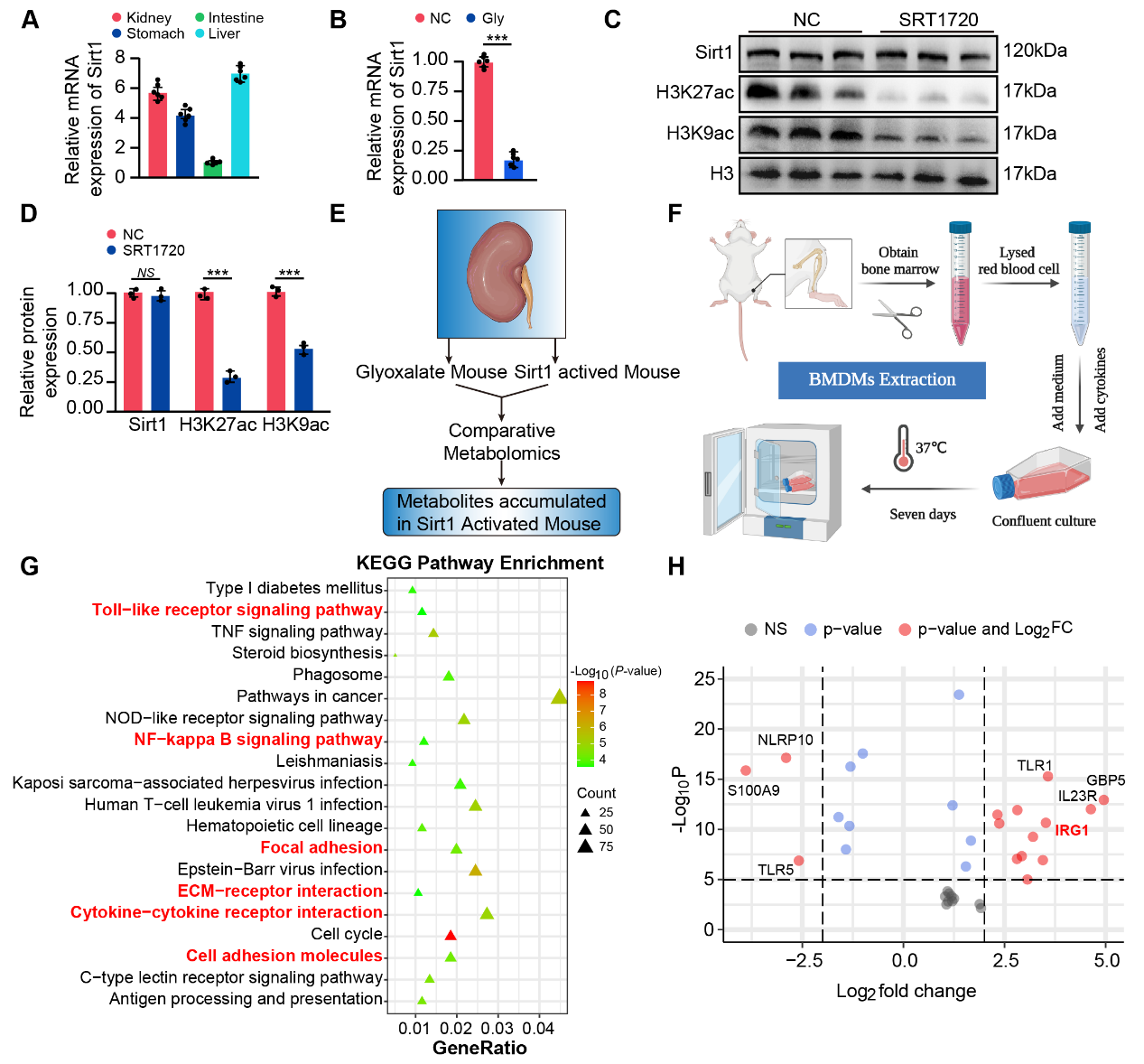


**Fig. S1** **Expression of Sirt1 in tissues and identification of differentially expressed genes.** (A) Relative mRNA analysis of the expression of Sirt1 in major metabolic organs, including the liver, kidney, intestine and stomach (n=6). (B) Relative mRNA analysis of Sirt1 levels in renal tissues of different treatments, including negative control (NC), and glyoxylate (Gly) (n=6). (C and D) Western blot analysis the effect of Sirt1 agonists on Sirt1 and acetylated histones (n=3). (E) GC-MS nontargeted metabolomic analysis was performed to assess the differential metabolites in the Gly group and Gly+SRT1720 group (n=6). (F) The process of BMDM acquisition. (G) KEGG enrichment analysis of the enrichment pathways of differentially expressed genes. (H) The volcano plot demonstrates the expression of 35 differentially expressed genes. ****P* < 0.001.

**
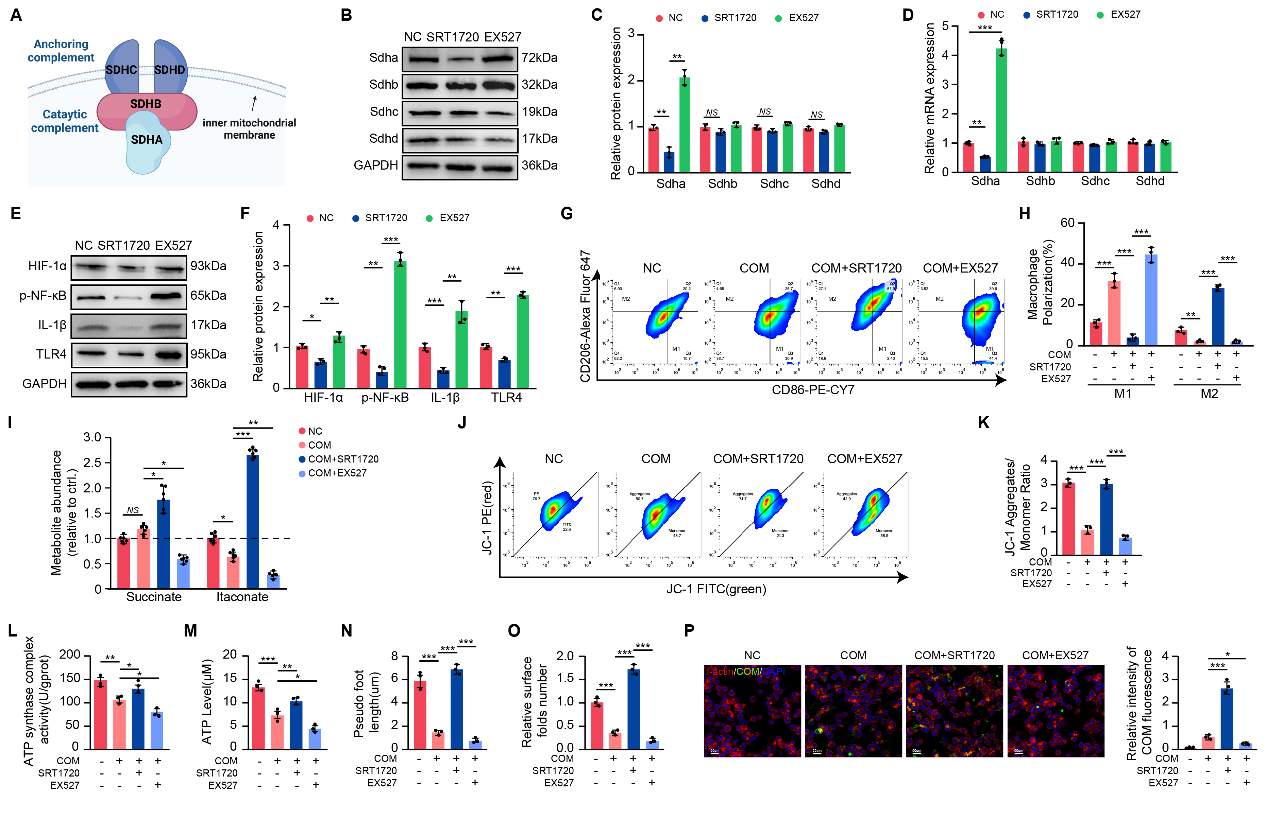
**

**Figure S2 Regulation of Sirt1 on downstream inflammatory genes.** (A) The structures of SDH, Sdha and Sdhb are catalytic subunits, and Sdhc and Sdhd are anchoring subunits. (B and C) Western blot and quantification of SDH subunits. (D) Relative mRNA analysis of SDH subunits and macrophage markers with Sirt1 alteration. (E and F) Western blot demonstrated the regulation of Sirt1 on inflammatory genes. (G and H) Flow cytometry analysis and quantification of the BMDM polarization state of F4/80^+^ cells with anti-CD86 and anti-CD206 antibodies. (I) Metabolic abundance of succinate and itaconate under Sirt1 activation/inhibition. (J and K) Flow cytometry demonstrated altered mitochondrial membrane potential under JC-1 dye. (L and M) ATP synthase activity and ATP levels of TECs. (N and O) Quantification of pseudopodia and surface folds in macrophages. (P) Fluorescence microscopy analysis of BMDM phagocytic ability (f-actin: red; COM: green; DAPI: blue). (Data are presented as mean ± SD, n = 3). **P* < 0.05, ***P* < 0.01, ****P* < 0.001.


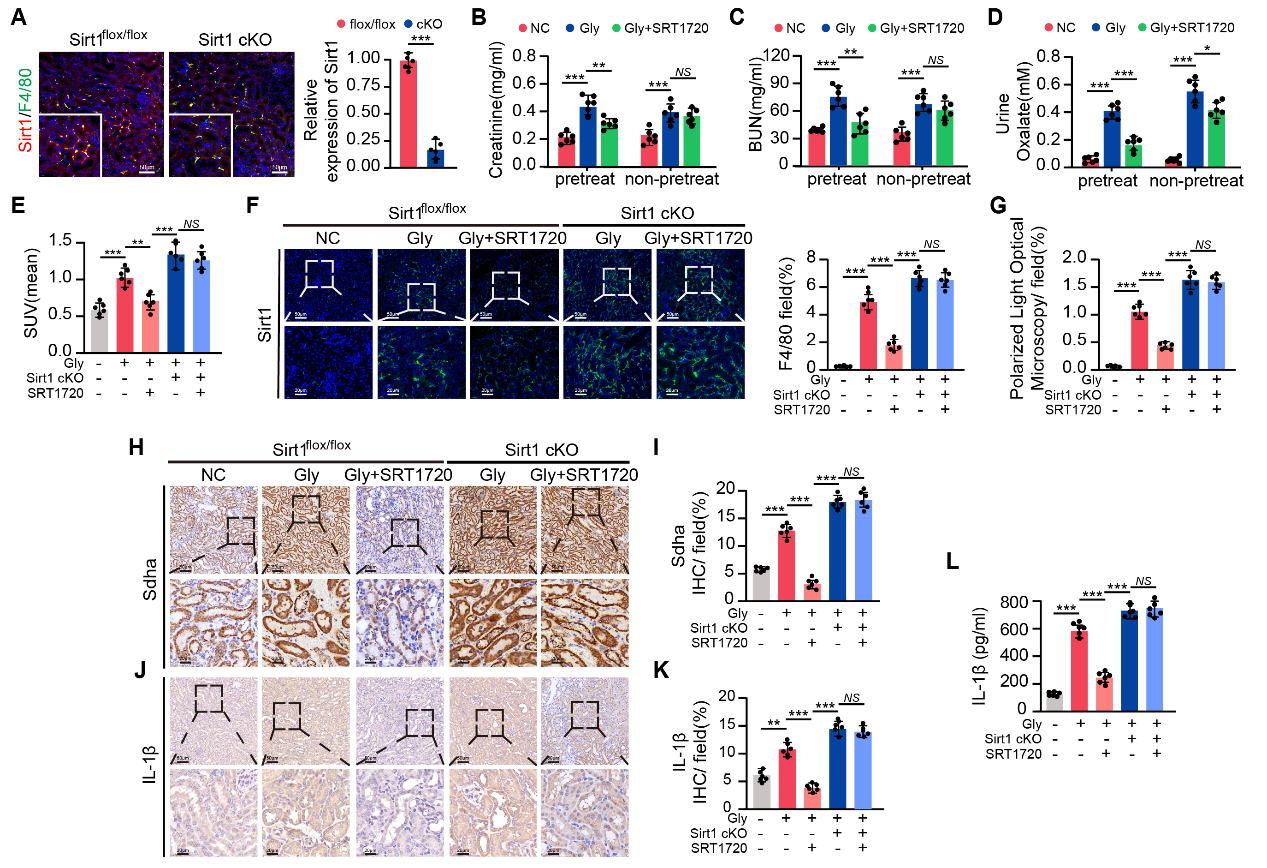


**Figure S3 Protective effect of Sirt1 on renal crystal deposition.** (A) IF showed Sirt1 (red) and macrophage marker F4/80 (green) of kidney tissues in Sirt1^flox/flox^ and Sirt1 cKO mice (200×, scale bar: 50 μm). (B and C) BUN and creatinine levels in mice pretreated three days ahead and non-pretreated. (D) Urine oxalate levels in mice pretreated three days ahead and non- pretreated. (E) The uptake value of ^18^F-FDG in the kidneys of different groups of mice. (F) IF showed macrophage marker F4/80 (green) of kidney tissues in Sirt1^flox/flox^ and Sirt1 cKO mice (200×, scale bar: 50 μm). (G) Polarized light optical microscopy showed CaOx crystal deposition in different groups. (H-K) IHC staining was used to measure Sdha and IL-1β in renal tissue (upper channel: 200×, scale bar: 50 μm; lower channel: 600×, scale bar: 20 μm). (L) IL-1β levels measured by ELISA in cell culture supernatants. (Data are presented as mean ± SD, n = 6). **P* < 0.05, ***P* < 0.01, ****P* < 0.001, *NS*: no significance.


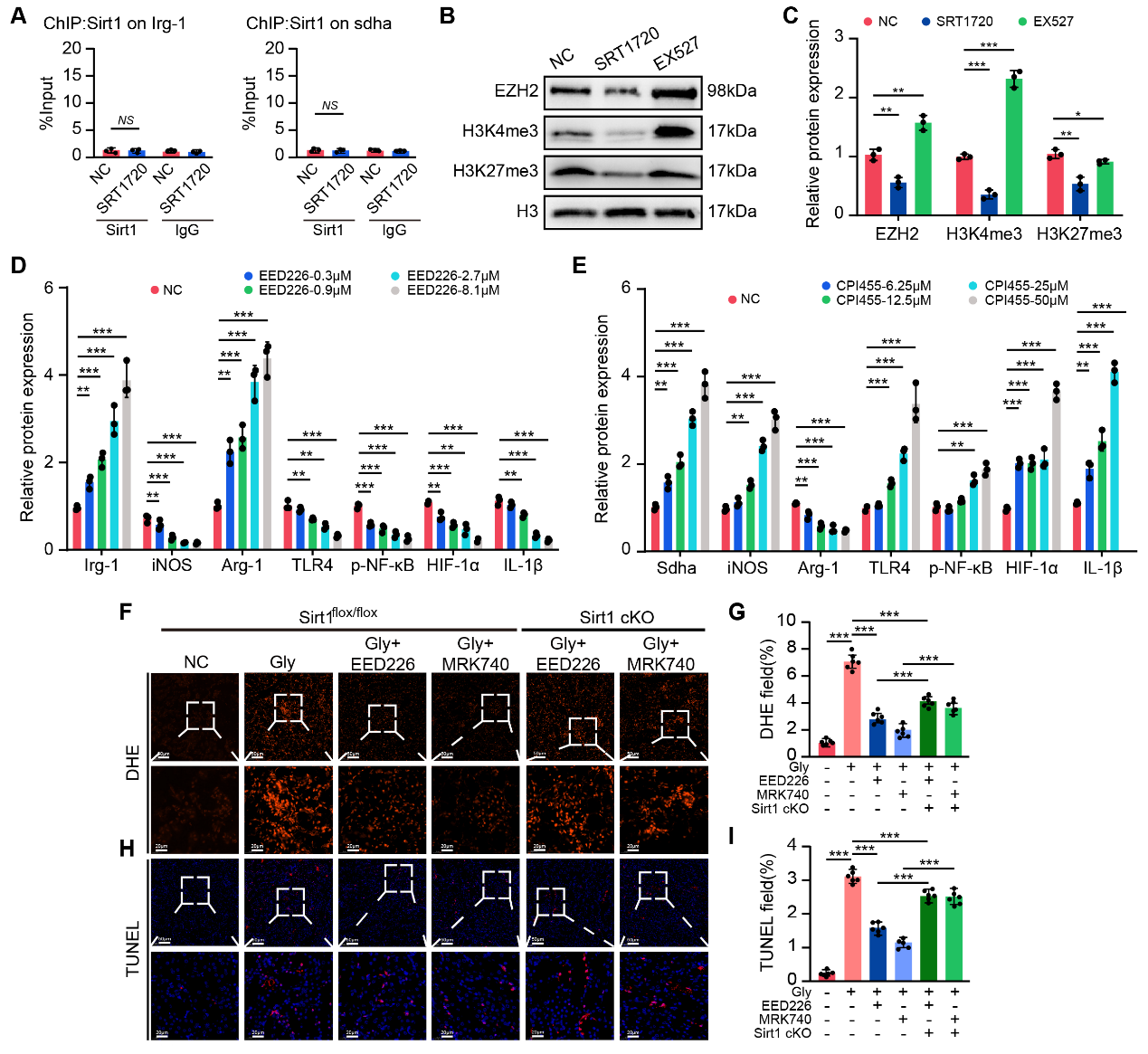


**Figure S4 Regulation of Sirt1 at the histone level.** (A) ChIP-qPCR analysis of Sirt1 binding to the Irg-1 and sdha promoter treated with SRT1720 (n=3). (B and C) Western blot showing EZH2, H3K27me3, and H3K4me3 levels in BMDMs (n=3). (D) The bar graph shows protein quantification of macrophage inflammation and polarization by inhibition of H3K27me3(n=3). (E) The bar graph shows protein quantification revealed the regulation of macrophage inflammation and polarization by activation of H3K4me3(n=3). (F-I) DHE and TUNEL staining revealed oxidative damage to TECs (upper channel: 200×, scale bar: 50 μm; lower channel: 600×, scale bar: 20 μm) (n=6). **P* < 0.05, ***P* < 0.01, ****P* < 0.001.


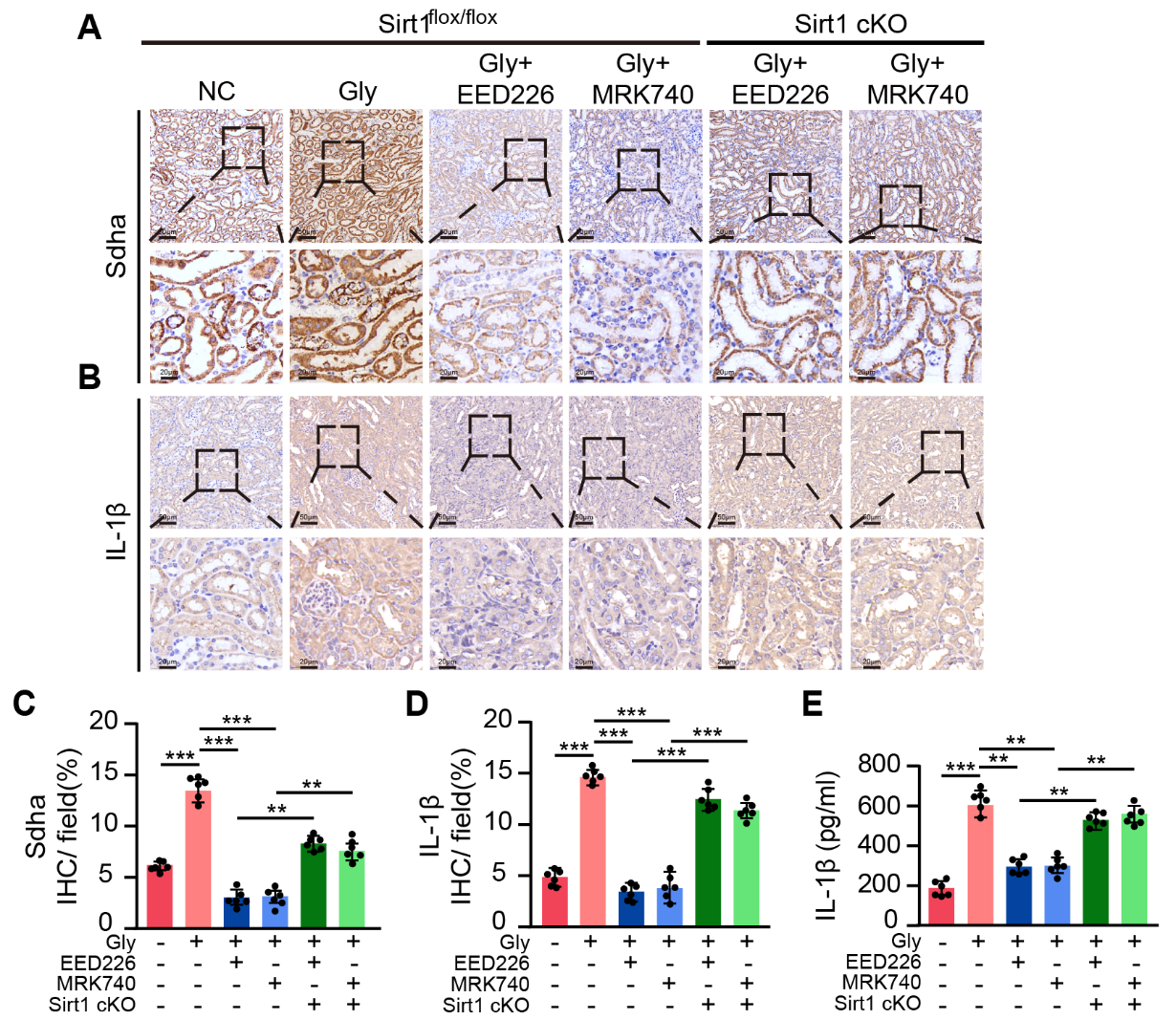


**Fig. S5 Protective effect of Sirt1 on renal crystal deposition via H3K27me3 and H3K4me3.** (A-D) IHC staining showed Sdha, and IL-1β levels in kidney tissue (200×, scale bar: 50 μm) (n=6). (E) IL-1β levels measured by ELISA in cell culture supernatants (n=6). **P* < 0.05, ***P* < 0.01, ****P* < 0.001, *NS*: no significance.


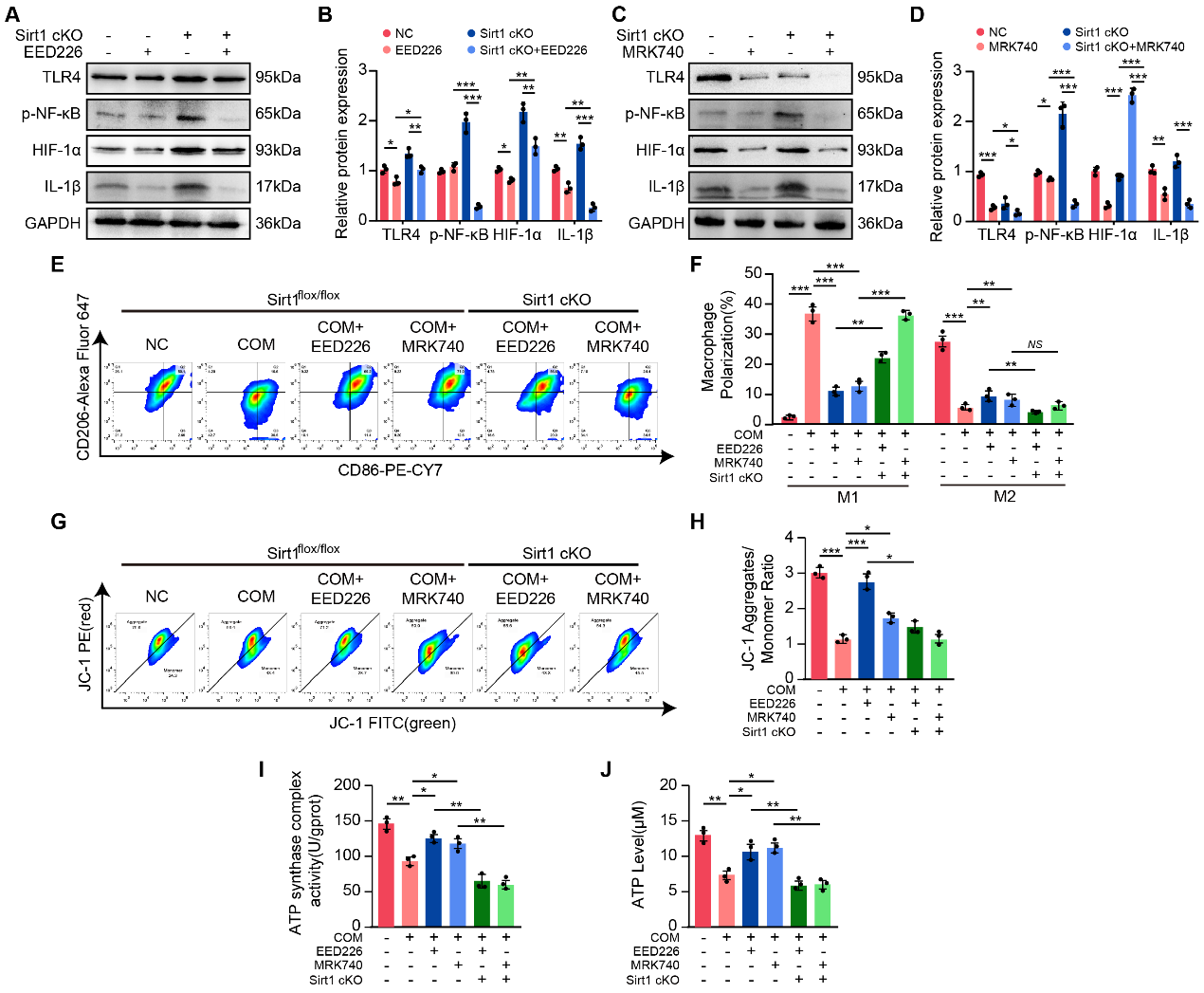


**Figure S6 Reversion experiments in macrophage polarization and TECs damage.** (A and B) Inhibition of Sirt1 concomitantly downregulated H3K27me3 on the protein levels of Irg-1 and downstream inflammatory genes analyzed by Western blot. (C and D) Inhibition of Sirt1 concomitantly downregulated H3K4me3 on the protein levels of Sdha and downstream inflammatory genes analyzed by Western blot. (E and F) Flow cytometric analysis and quantification of the BMDM polarization state of F4/80^+^ cells with anti-CD86 and anti-CD206 antibodies. (G and H) Flow cytometry demonstrated mitochondrial membrane potential changes with JC-1 dye. (I and J) ATP synthase activity and ATP levels of TECs. Data are presented as mean ± SD, n = 3). **P* < 0.05, ***P* < 0.01, ****P* < 0.001.


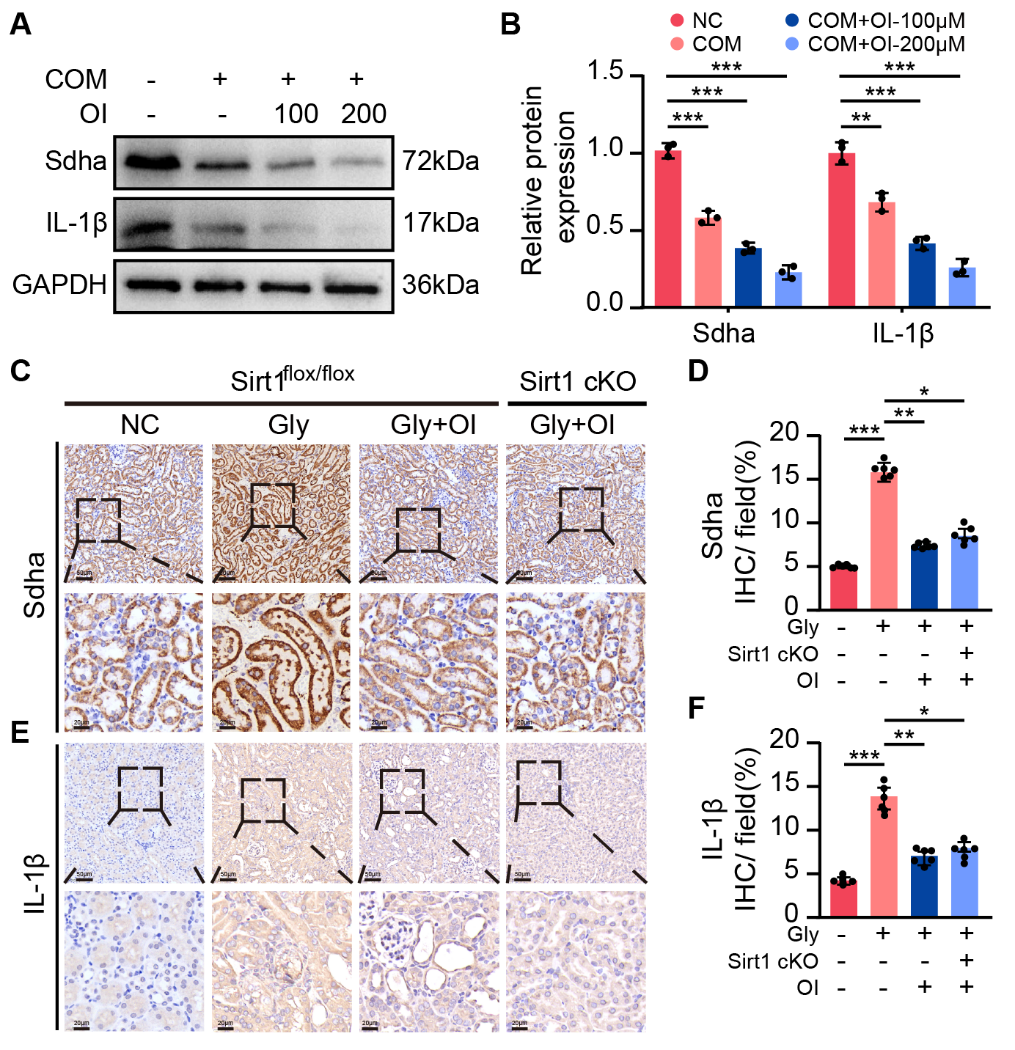


**Fig. S7 OI reduces crystal deposition and Sdha levels.** (A and B) Western blot showing the Sdha level under OI treatment in BMDMs (n=3). (C-F) IHC staining showed Sdha and IL-1β levels in kidney tissue (200×, scale bar: 50 μm) (n=6). **P* < 0.05, ***P* < 0.01, ****P* < 0.001.

**Supplemental Tables**

**Supplemental Table 1. Plasma BUN, creatinine and urine oxalate in mice.**

| **Group** | **BUN**  **(mg/ml)** | **Creatinine**  **(mg/ml)** | **Urine Oxalate**  **(****mM)** |
| --- | --- | --- | --- |
| NC | 36.382±1.532 | 0.251±0.076 | 0.032±0.011 |
| Gly | 68.374±9.844 ^a^ | 0.419±0.089 ^a^ | 0.395±0.053 ^a^ |
| Gly+SRT1720 | 43.965±6.938 ^b^ | 0.321±0.087 ^b^ | 0.126±0.042 ^b^ |
| Gly+cKO | 87.623±17.333 ^b^ | 0.594±0.091 ^b^ | 0.546±0.095 ^b^ |
| Gly+SRT1720+cKO | 89.963±21.527 ^c^ | 0.568±0.103 ^c^ | 0.583±0.093 ^c^ |
|  |  |  |  |
| NC | 28.042±5.486 | 0.167±0.038 | 0.055±0.007 |
| Gly | 64.453±9.748 ^a^ | 0.538±0.074 ^a^ | 0.382±0.073 ^a^ |
| Gly+OI | 38.012±7.567 ^b^ | 0.292±0.058 ^b^ | 0.278±0.042 ^b^ |
| Gly+OI+cKO | 46.374±9.826 ^b^ | 0.343±0.067 ^b^ | 0.249±0.081 ^b^ |

Abbreviations: BUN, blood urea nitrogen; NC, normal control; Gly, glyoxylate; OI, 4-octyl itaconate; cKO, Sirt1 cKO mice.

^a^*P*<0.05 compare with the NC group

^b^*P*<0.05 compare with the Gly group

^c^*P*<0.05 compare with the Gly+cKO group

**Supplementary Table S2. Primer sequences used for the real-time qPCR analysis.**

| **Primer** | **Name** | **Primer sequence (5′ - 3′)** |
| --- | --- | --- |
| qRT‒PCR | Sirt1 | F: ATGACGCTGTGGCAGATTGTT  R: CCGCAAGGCGAGCATAGAT |
|  | Irg-1 | F: GGTGAGCCTGCAACTTGGAT  R: CGGATCTTCTGTCCGATCTGC |
|  | Sdha | F: GCGGTGGTCACCTTGATCC  R: CCTCTGTAGAAGCGTCTGAATG |
|  | iNOS | F: CACCTTGGAGTTCACCCAGT  R: ACCACTCGTACTTGGGATGC |
|  | Arg-1 | F: TGGCTTGCGAGACGTAGAC  R: GCTCAGGTGAATCGGCCTTTT |
|  | TLR4 | F: ATGGCATGGCTTACACCACC  R: GAGGCCAATTTTGTCTCCACA |
|  | HIF-1α | F: GTCCCAGCTACGAAGTTACAGC  R: CAGTGCAGGATACACAAGGTTT |
|  | IL-1β | F: TTCAGGCAGGCAGTATCACTC  R: GAAGGTCCACGGGAAAGACAC |
|  | GAPDH | F: TGGTGAAGGTCGGTGTGAAC  R: GCTCCTGGAAGATGGTGATGG |
| ChIP‒qPCR | Irg-1 | F: AGGGTGCCTAGCCCATAAGT  R: AGCCCCTAACAGCGTTCAAA |
|  | Sdha | F: TCCTGCACAGCGACAAGAAG  R: TACAGCTTGGCCCCAAAGAG |

**Supplementary Table S3. Antibodies used in the manuscript.**

| **Name (Supplier name, Catalogue number)** | **RRID** |
| --- | --- |
| Sdhb (proteintech, 10620-1-AP) | RRID: AB_2285522 |
| Sdhc (proteintech, 14575-1-AP) | RRID: AB_2183291 |
| iNOS (proteintech ,80517-1-RR) | RRID: AB_2918898 |
| TLR4 (proteintech, 66350-1-Ig) | RRID: AB_2881730 |
| EZH2 (proteintech, 21800-1-AP) | RRID: AB_10858790 |
| H3K9ac (proteintech, 29133-1-AP) | RRID: AB_3085344 |
| H3 (proteintech, 17168-1-AP) | RRID: AB_2716755 |
| Sdhd (abclone, A16240) | RRID: AB_2772166 |
| GAPDH (abclone, AC002) | RRID: AB_2736879 |
| Arg-1 (abclone, A1847) | RRID: AB_2763883 |
| H3K27ac (abclone, A7253) | RRID: AB_2767797 |
| IL-1β (CST, 12242) | RRID: AB_2715503 |
| Sirt1 (CST, 8469) | RRID: AB_10999470 |
| Sirt1 (Sigma, 04-1557) | RRID: AB_1977495 |
| Irg-1(CST, 17850) | RRID: AB_3064865 |
| Sdha (CST, 11998) | RRID: AB_2750900 |
| HIF-1α (CST, 14179) | RRID: AB_2622225 |
| NF-κb p65 (CST, 8242) | RRID: AB_10859369 |
| Anti-mouse IgG, HRP-linked Antibody (CST, 7076) | RRID: AB_330924 |
| Anti-rabbit IgG, HRP-linked Antibody (CST, 7074) | RRID: AB_2099233 |
| PE Rat anti-Mouse F4/80 (BD bioscience, 565410) | RRID: AB_2687527 |
| FITC Rat anti-CD11b (BD bioscience, 561688) | RRID: AB_10898180 |
| PE-CyTM7 Rat anti-Mouse CD86 (BD bioscience, 560582) | RRID: AB_1727518 |
| [Alexa Fluor® 647 Rat Anti-Mouse CD206](https://www.bdbiosciences.com/zh-cn/products/reagents/flow-cytometry-reagents/research-reagents/single-color-antibodies-ruo/alexa-fluor-647-rat-anti-mouse-cd206.565250) (BD bioscience, 568808) | RRID: AB_3094491 |
